# Serum proteins and faecal microbiota as potential biomarkers in newly diagnosed, treatment-naïve inflammatory bowel disease and irritable bowel syndrome patients

**DOI:** 10.1007/s00109-025-02558-5

**Published:** 2025-06-07

**Authors:** Mario Matijašić, Anja Barešić, Hana Čipčić Paljetak, Mihaela Perić, Marina Panek, Ana Kunović, Dina Ljubas Kelečić, Darija Vranešić Bender, Katja Grubelić Ravić, Dunja Rogić, Margareta Antolic, Ivana Horvat, Ivana Kraljević, Marko Banić, Željko Krznarić, Donatella Verbanac

**Affiliations:** 1 Center for Translational and Clinical Research, University of Zagreb School of Medicine, Zagreb, Croatia; 2 Laboratory for Computational Biology and Translational Medicine , Ruđer Bošković Institute, Zagreb, Croatia; 3 University of Zagreb School of Medicine, Zagreb, Croatia; 4 Department of Internal Medicine, Unit of Clinical Nutrition, University Hospital Centre Zagreb, Zagreb, Croatia; 5 Department of Internal Medicine, Division of Gastroenterology and Hepatology, University Hospital Centre Zagreb, Zagreb, Croatia; 6 Department of Laboratory Diagnostics, University Hospital Centre Zagreb, Zagreb, Croatia; 7 Department of Endocrinology, University Hospital Centre Zagreb, Zagreb, Croatia; 8 Department of Gastroenterology, Hepatology and Clinical Nutrition, University Hospital Dubrava, Zagreb, Croatia; 9 Faculty of Pharmacy and Biochemistry, University of Zagreb, Zagreb, Croatia

**Keywords:** Inflammatory bowel disease, Crohn’s disease, Ulcerative colitis, Irritable bowel syndrome, Serum biomarkers, Faecal microbiota, Treatment-naïve patients

## Abstract

**Abstract:**

Molecular biomarkers are valuable tools to predict the disease and determine its course. Several markers have been associated with inflammatory bowel disease (IBD) and irritable bowel syndrome (IBS); however, none is sufficiently reliable to enable accurate diagnosis. We characterized a broad panel of serum proteins to assess disease-specific biomarker profiles and associate these findings with faecal microbiota composition in newly diagnosed IBD and IBS patients and healthy individuals. The study cohort consisted of 49 newly diagnosed treatment-naïve adult patients (13 Crohn’s disease (CD), 13 ulcerative colitis (UC), and 23 IBS) and 12 healthy individuals. Inflammatory and metabolism-related serum proteins were assessed using PEA multiplex panels, while gut microbiota composition was determined by 16 s rRNA gene amplicon sequencing. Serum proteins AXIN1, TNFSF14, RNASE3, EN-RAGE, OSM, ST1A1, CA13 and NADK were identified as markers with the most promising specificity/sensitivity and predictivity between healthy and disease groups, while IL-17A and TNFRSF9 enabled differentiation between IBD and IBS patients. Increased abundance of *Enterobacteriaceae* was associated with protein markers significantly elevated in IBD/IBS. In contrast, depletion of beneficial taxa like *Ruminococcaceae* and *Verucomicrobiaceae* (i.e. *Akkermansia muciniphila*) was associated with decrease of a set of markers in diseased groups. Differences in the abundance of *Turicibacteriaceae* were more predictive to discern CD from UC than any of the serum proteins investigated. By using a broad panel of inflammation and metabolism-related proteins, we determined serum markers with significantly different levels in treatment-naïve IBD and IBS patients compared to healthy individuals, as well as between IBD and IBS.

****Key messages**:**

- Significant changes in the levels of several serum proteins and abundances of faecal bacterial taxa between study groups were found.
- Increased levels of AXIN1, TNFSF14, RNASE3, EN-RAGE, OSM, ST1A1, CA13 and NADK characterize both IBD and IBS, while IL-17A and TNFRSF9 differentiate IBD from IBS.
- Increase of *Enterobacteriaceae* and depletion of beneficial taxa *Ruminococcaceae* and *Verucomicrobiaceae* (i.e. *Akkermansia muciniphila*) was found in IBD and IBS. Differences in *Turicibacteriaceae* were more predictive to discern CD from UC than any of the serum proteins investigated.

**Supplementary Information:**

The online version contains supplementary material available at 10.1007/s00109-025-02558-5.

## Introduction

Inflammatory bowel disease (IBD) with its two clinically and morphologically different entities, ulcerative colitis (UC) and Crohn’s disease (CD), occurs in genetically susceptible host with aberrant immune response towards altered gut microbiota [1]. Aetiology of irritable bowel syndrome (IBS) includes dietary, immunological, inflammatory, neurological, genetic and environmental factors, as well as gut microbiota dysbiosis [2]. Both diseases are highly prevalent, have a significant negative impact on patients’ quality of life and present a substantial economic burden on healthcare systems [3, 4].

IBD and IBS can manifest with similar symptoms. Diagnostic procedures include evaluation of clinical and laboratory findings, combined with histological examination of mucosal biopsies which remains the gold standard for clinical diagnosis. Biomarkers, obtained using less invasive techniques, are helpful tools in both IBD diagnosis and monitoring the disease activity, with serum C-reactive protein (CRP) and faecal calprotectin routinely used in clinical practice. Although superior to CRP in terms of sensitivity and specificity, faecal calprotectin has its limitations as a marker and cannot be considered as a reliable alternative to colonoscopy [5]. Thus, there is an unmet need for identifying new disease-specific non-invasive biomarkers, which would contribute to characterization of the disease and stratification of patients and enable personalized treatment options. Recent research focused on identifying inflammatory protein profiles in the serum of IBD and IBS patients, often combining several biomarkers or using multiplex biomarker panels to achieve a higher power of discrimination [6–9]. As IBD and IBS-related dysbiosis usually associates with depletion of beneficial and increase of pro-inflammatory bacteria, an emerging number of reports explored gut microbiota constituents as possible biomarkers [10]. In this study, we characterized a broad panel of inflammatory and metabolism-related serum proteins to determine disease-specific biomarker profiles in newly diagnosed and treatment-naïve IBD (CD and UC) and IBS adult patients and correlated these findings to faecal microbiota profiles of patients and healthy individuals.

## Materials and methods

### Study population

The study was conducted at the Center for Translational and Clinical Research, University of Zagreb School of Medicine (UZSM), and the Department of Gastroenterology, University Hospital Center Zagreb (UHCZ), on adult participants with suspected IBD between 2015 and 2018 and on healthy volunteers (Table 1). IBD was diagnosed following thorough clinical, endoscopic and histological evaluation and categorized according to the Montreal classification. Disease severity was assessed according to Simple Endoscopic Score for Crohn Disease (SES-CD) and Mayo Endoscopic Score (ES) for UC [11]. IBD patients’ treatment-naivety was defined as no exposure to any IBD-related medical therapies (5-ASA, corticosteroids, immunomodulators and biologics) prior to sampling. IBS was diagnosed according to Rome III criteria [12]. No information on IBS subtypes was recorded during recruitment. Participants were > 18 years old, did not have chronic, malignant or autoimmune diseases and were not pregnant. The enrolment in the study did not affect or delay treatment initiation. Healthy control subjects had no history of gastrointestinal or other chronic disorders or reported current GI symptoms. None of the participants received immunosuppressive or antibiotic treatment 3 months before sample collection or reported use of probiotics.

**Table 1:** Demographic data and biochemical and faecal markers for study participants

*n*	CD	UC	IBS^§^	Healthy
	13	13	23	12
Demographic data				
Median age at diagnosis (range)	45 (21–72)	32 (18–54)	32 (19–56)	35 (24–56)
Female, *n* (%)	9 (69)	7 (54)	13 (57)	6 (50)
BMI median (range)	24 (20–32)	23.1 (18–32)	22.6 (19–33)	24.6 (23–29)
Biochemical parameters				
CRP, mg/L median (range)	2.2 (< 0.3–36.8)	0.7 (< 0.3–17.5)	0.6 (< 0.3–18.5)	
Faecal calprotectin mg/kg median (range)^#^	88 (< 20–348)	743 (21–1800 +)	30 (< 20–373)	
Disease severity*				
Mild	7	5		
Moderate/severe	4/1	8/0		
Unknown	1			
Montreal classification				
Age at diagnosis				
A1 < 17 years	0	0		
A2 17–40 years	5	10		
A3 > 40 years	8	3		
Location/extension				
L1 ileal^a^/E1 proctitis^b^	5	8		
L2 colonic^a^/E2 left-sided colitis^b^	4	1		
L3 ileocolonic^a^/E3 extensive colitis^b^	3	4		
Unknown^†^	1			
Behaviour CD				
B1 non-stricturing, non-penetrating	7			
B2 stricturing	3			
B3 penetrating	0			
B2p perianal modifier	1			
B3p perianal modifier	1			
Unknown^†^	1			

^*^Disease severity at the diagnosis was assessed according to SES endoscopic score for CD and Mayo score for UC [11]. SES-CD score, inactive ≤ 2 pts, mild 3–6 pts, moderate 7–15 pts, severe ≥ 16 pts. Mayo ES score, inactive 0 pts, mild 1 pts, moderate 2 pts, severe 3 pts

^§^IBS was diagnosed according to Rome III criteria [12]

^#^Missing data in 38%, 23% and 52% of CD, UC and IBS patients, respectively

^†^Location and behaviour were determined during endoscopy, and one patient had no inflamed sites but was subsequently diagnosed as CD

^a^CD

^b^UC

The study was approved by the competent institutional ethics committees (380–59-10,106–14–55/149, 641–01/14–02/01; 02/21/JG, 8.1.−14/45–2) and conducted in accordance with the Declaration of Helsinki. All participants received the necessary information on the study and provided signed informed consent. Participants’ data were available only to the attending physician, while the collected data were pseudoanonymized and stored electronically, and researchers fully complied with prescribed procedures for personal data protection.

### Protein profiling

Blood acquired before any medical procedure or treatment was immediately processed by centrifugation to obtain serum, which was aliquoted and stored at − 80 °C. A total of 92 inflammation-related and 92 metabolism-associated proteins were analyzed using Proseek Multiplex Inflammation and Proseek Multiplex Metabolism panel (Olink Proteomics, Sweden). Proseek uses proximity extension assay (PEA) technology [6] for quantifying serum proteins, reported as log2 normalized values (normalized protein expression, NPX), corresponding to relative protein levels in the sample. Values below the level of detection (LOD) were replaced with LOD, following manufacturer’s recommendation. Proteins with > 40% of values below LOD were excluded from further analyses (Supplementary Table S1 and S2).

### Faecal microbiota profiling

The set of faecal samples consisted of those collected from the participants in Čipčić Paljetak study [13], who also provided blood samples, with the inclusion of additional participants based on the faecal/blood sample availability.

The faecal sample collection, DNA extraction, sequencing and microbiota characterization utilize the same protocol as in our previous study [13]. Briefly, faecal samples were self-collected by participants prior to diagnostic colonoscopy procedure using OMNIgene.GUT faecal kit (DNA Genotek, Canada) and processed within 7 days after collection. Faecal DNA was extracted using Fast DNA SPIN Kit for Faeces (MP Biomedicals, USA). The DNA quantity and purity were determined based on the absorbance and fluorescence measurements using Nanodrop 2000 and Qubit 3.0, respectively, both Thermo Fisher Scientific, Germany. DNA integrity was confirmed by agarose gel electrophoresis.

Faecal bacterial communities were profiled by V3-V4 regions of 16S rRNA gene amplicon sequencing using MiSeq platform (Illumina, USA).

Raw sequencing files were processed using QIIME pipeline [14]. Operational Taxonomic Units (OTUs) at phylum to genus taxonomy levels were assigned using usearch and PyNast alignment against the GreenGenes database (v13_8). Results are presented at the family level, but sufficient sequencing depth allowed discrimination at the species level for some taxa, i.e. *Akkermansia muciniphila*, *Haemophilus parainfluenzae* and *Faecalibacterium prausnitzii*. To identify differentially abundant taxa and calculate the taxon-level effect size of the difference between groups, ALDEx2 (ANOVA-like differential expression analysis) R package was used [15].

### Statistical analyses

Differences between groups were assessed using Kruskal–Wallis test, and all biomarkers with unadjusted *p*-value > 0.05 were removed from subsequent analyses. Significance levels of FDR-adjusted pairwise Wilcoxon test were reported for various pairwise comparisons of groups.

Heatmaps are based on the effect sizes of each taxon as calculated by the ALDEx2 R package for bacterial taxa and Cohen’s *D* effect sizes for protein biomarkers. For the rationale on using effect sizes as robust measure of differential abundances in the context of compositional nature of these data, please consider Čipčić Paljetak et al. and references therein [13]. Cohen’s thresholds of < 0.147, 0.33 and 0.474 were defined as negligible, small and medium effect sizes, respectively, with > 0.474 interpreted as large effect size (also applied to the respective negative values). Clusters were determined using k-means clustering, as implemented in R (hclust, method = “ward”), and the optimal number of clusters chosen upon manual inspection. Receiver operating characteristic (ROC) and area under the ROC curve (AUC) were calculated using ROCit package in R, with maximum Youden index (J) defined as max true positive rate against false positive rate (TPR-FPR) used as the summary statistic.

Correlation is reported as Spearman’s rho coefficient as implemented in R (cor, use = “complete.obs”). Correlation matrices are made using corrplot package, with hierarchically clustered biomarkers using “order = hclust” option.

## Results

The study cohort consisted of 61 participants classified into four groups: 13 diagnosed with Crohn’s disease (CD), 13 with ulcerative colitis (UC), 23 with irritable bowel syndrome (IBS) and 12 healthy subjects (H) (Table 1). The median age of CD patients was higher than for other disease groups (45 years vs. 32, respectively), with a higher proportion of female participants (69%).

### Differential levels of serum biomarkers

Out of 184 serum proteins investigated, 151 were successfully quantified, while 33 were excluded from further analyses (Supplementary Tables S1 and S2). Differences in protein levels between groups, identified using univariate analyses, are listed in Supplementary Table S3 and their median values provided in Supplementary Table S4. Along with comparing levels between individual groups, comparisons with IBD group (UC + CD) were performed. Overall, 55 proteins showed statistically significant differences in serum levels between groups (corrected *p*-values < 0.05).

**Fig. 1:**
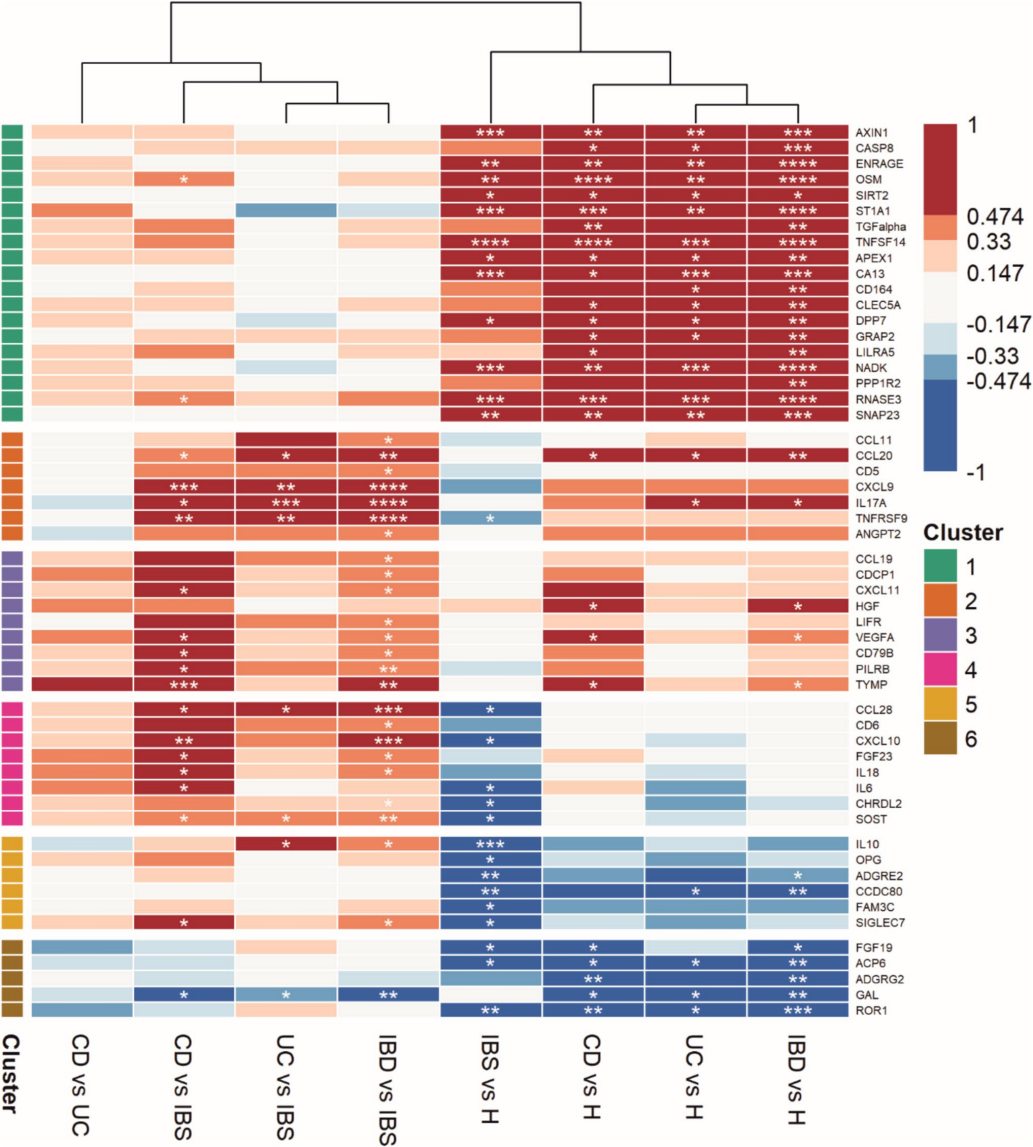
Heatmap of serum protein biomarkers effect sizes in pairwise comparisons of study groups. K-means clustering with six clusters. For easier heatmap navigation, statistical significance was added to corresponding cells as FDR-corrected *p*-value (* < 0.05, ** < 0.01, *** < 0.001, **** < 0.0001)

Calculated effect sizes (Supplementary Table S5) were clustered and proteins with similar effect sizes across the comparisons are represented in Fig. 1.

The heatmap demonstrated a clear discrimination between healthy and IBD/IBS disease groups (columns 5–8), as well as between IBD and IBS (columns 2–4). None of the proteins statistically discriminated CD from UC (column 1), albeit some had medium/large effect sizes.

The most profound difference was observed between healthy and disease groups (clusters 1, 5 and 6). Proteins in cluster 1 were significantly increased in CD/UC/IBS patients, offering clear discrimination from healthy subjects. All but two of the proteins in clusters 2, 3 and 4 significantly differentiate IBD from IBS, most profoundly CXCL9, IL-17 A and TNFRSF9 (*p* < 0.0001), as well as CCL28 and CXCL10 (*p* < 0.001). Additionally, levels of IL-17 A were significantly increased in UC compared to healthy individuals, while levels of CXCL9 and TNFRSF9 were reduced in IBS. Proteins in cluster 5 showed lower levels in the IBS than in the healthy group, most significantly IL-10 (*p* < 0.001). Although the moderate/large effect sizes suggested lower concentration of these markers in CD and UC compared to the healthy group, the majority of observed trends were not statistically relevant. Finally, cluster 6 contained proteins decreased in IBD/IBS compared to healthy individuals. The most significant depletion was identified for ROR1 (*p* < 0.001). In contrast to the other proteins in this cluster, GAL was decreased in IBD compared to both healthy and IBS groups (*p* < 0.01).

### Faecal microbiota composition and clustering with biomarkers

**Fig. 2:**
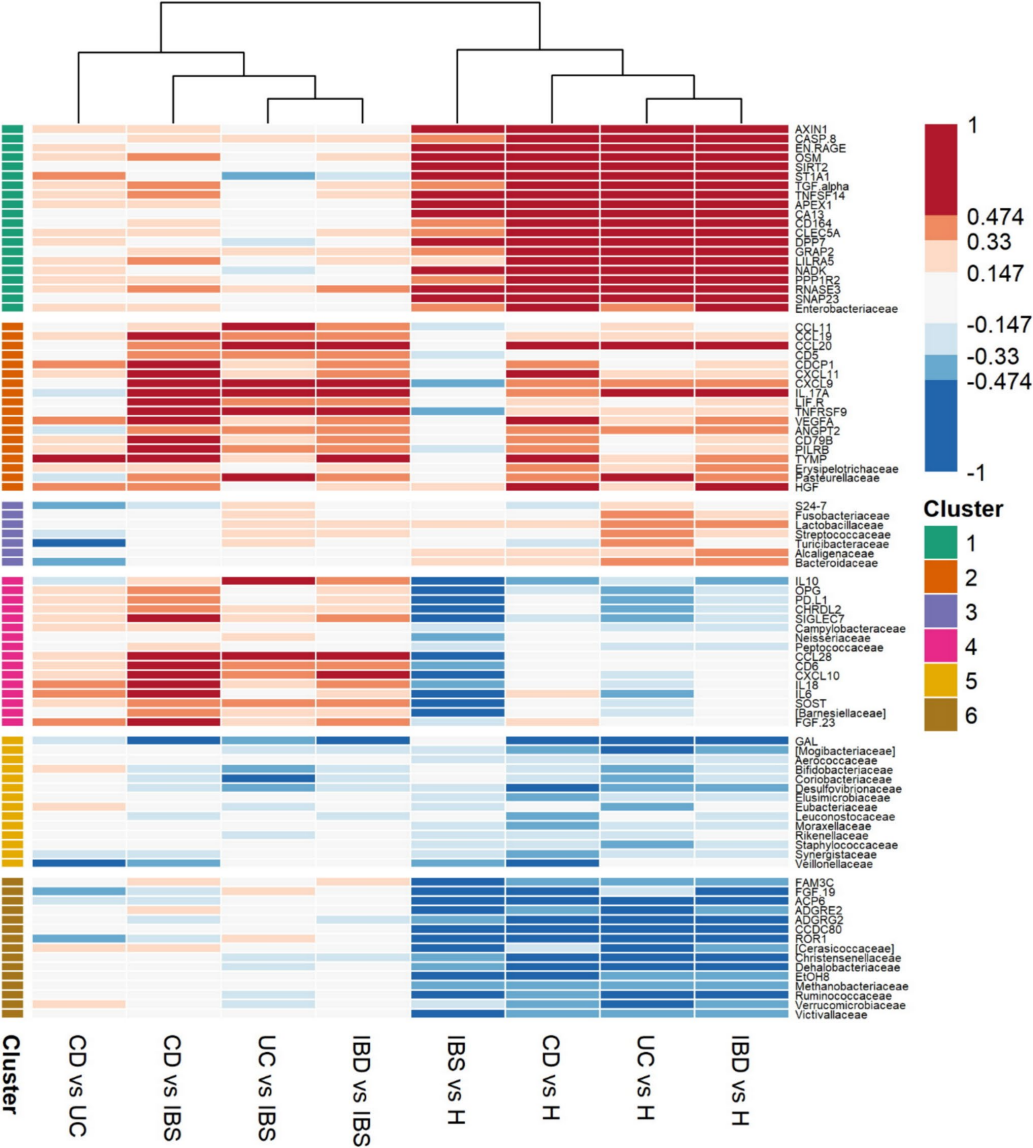
Heatmap of serum protein biomarkers and faecal bacteria effect sizes in pairwise comparisons of study groups. K-means clustering with six clusters

To assess whether serum protein levels could be associated with microbiota, bacterial composition in faecal samples was determined and effect sizes between study groups calculated (Supplementary Table S6). A set of 35 differentially abundant bacterial families present in all subject groups, with the effect sizes > 0.3 in any of the group comparisons, was chosen for the association with serum protein data (Fig. 2).

*Enterobacteriaceae*, highly abundant in faeces of IBD and IBS patients, grouped with proteins elevated in disease states (cluster 1), while *Erysipelotrichaceae* and *Pasteurellaceae*, increased in CD and UC, associated with proteins differentiating IBD from IBS (cluster 2). Families depleted in patients (e.g. *Christensenellaceae*, *Ruminococcaceae* and *Verrucomicrobiaceae*) clustered with proteins with lower levels in both IBD and IBS (cluster 6). Clusters 3 and 5 grouped bacteria not associated with serum proteins (except GAL). Although in different clusters, *Turicibacteriaceae* and *Veillonellaceae* were more abundant in UC individuals and offered differentiation between CD and UC with high effect sizes.

### ROC performance of individual biomarkers

**Fig. 3:**
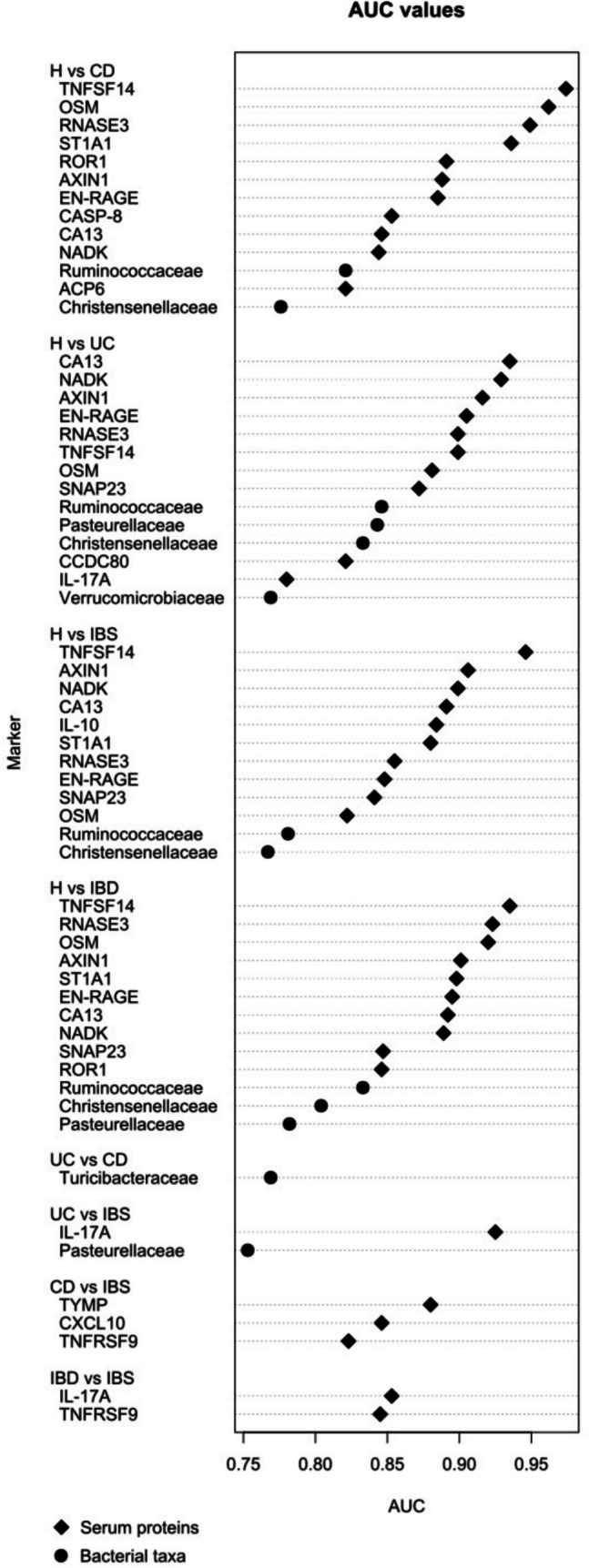
Study group differentiation based on the AUC values (ROC) for selected serum proteins and faecal bacterial taxa based on Youden index (*J* > 0.65, *J* > 0.45, respectively)

Given the differences between study groups, the predictive power of serum and bacterial markers as potential discriminators between the IBD, IBS and healthy individuals was investigated. The performance of selected serum proteins (*p* < 0.001 in any of the comparisons) and relevant bacterial taxa (> 0.30 effect size in any of the pairwise comparisons) in terms of receiver operating characteristic (ROC) curves is provided in Supplementary Table S7. The AUC for a selection of serum proteins (*J* > 0.65) and faecal bacterial taxa (*J* > 0.45) is depicted in Fig. 3.

**Fig. 4:**
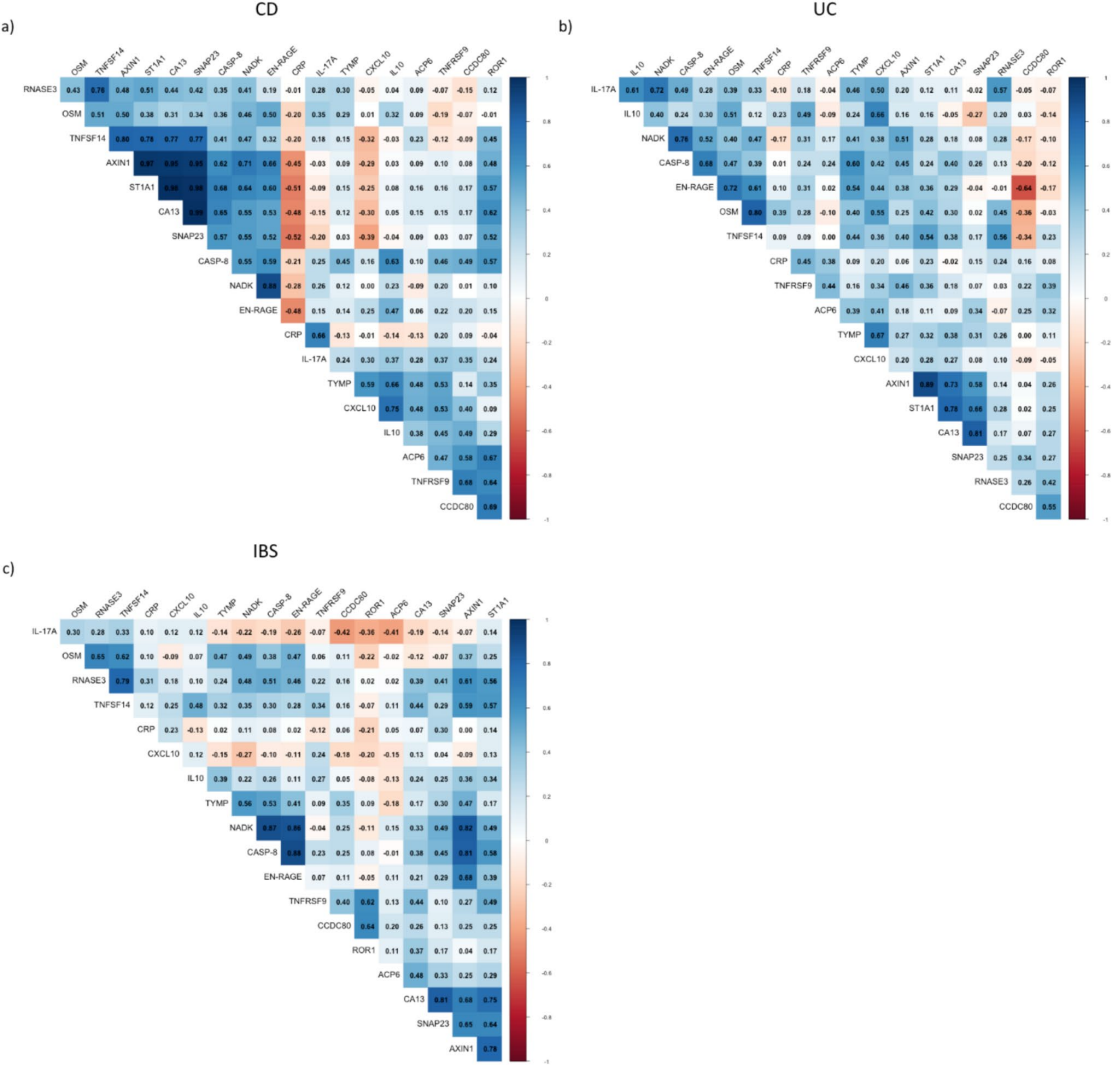
Spearman correlation matrices of selected serum proteins and CRP for **a** CD, **b** UC and **c** IBS group

Median values of CRP with standard deviations per study group, as well as statistical significance, J-index and AUC values between groups, are given in Supplementary Table S8. Faecal calprotectin could not be incorporated in this analysis due to large portion of missing values, as reported in Table 1. Spearman correlation between selected serum proteins (Fig. 4) with addition of CRP was performed, comparing patients within IBS, CD and UC categories of diagnoses separately, and shown in Fig. 4.

## Discussion

The crucial obstacle to guide personalized management of IBD and IBS from their diagnosis onward is the lack of sensitive and specific disease biomarkers. Although several markers have been associated with IBD and IBS, none is suitable for direct clinical practice or ensure safe and effective disease management. This study assessed a broad panel of serum proteins to evaluate disease-specific profiles in a cohort of newly diagnosed, treatment-naïve adult IBD (CD and UC) and IBS patients and to associate these findings to faecal microbiota composition of patients and healthy individuals.

This research reports clear differentiation between healthy and diseased individuals, as well as between IBD and IBS patients based on protein biomarker profiles. Expectedly, the most notable difference was observed between healthy and IBD/IBS groups, with the majority of proteins significantly increased in IBD/IBS individuals. Most of these proteins were previously implicated in IBD pathogenesis: EN-RAGE (extracellular newly identified receptor for advanced glycation end products binding protein (S100 A12)) [16], OSM (oncostatin M) [17], TNFSF14 (tumour necrosis factor superfamily member 14 (LIGHT))[18] and RNASE3 (eosinophil cationic protein) [19]. While discerning IBD/IBS patients from healthy population, these proteins do not distinguish CD from UC or easily discriminate between IBD and IBS [20]. We identified elevated levels of AXIN1 (axin1) and CASP-8 (caspase 8), proteins associated with intestinal inflammation and carcinogenesis [21, 22], as well as an increase of ST1 A1 (sulfotransferase 1 A1) in IBD group, which correlates with a recent report employing PEA multiplex technology [9]. Interestingly, an increase in IBD/IBS was also observed for CA13 (carbonic anhydrase XIII), protein associated with colorectal cancer (CRC) [23], as well as SNAP23 (synaptosomal-associated protein 23) and NADK (NAD kinase) which are linked to insulin signalling and type 2 diabetes mellitus (T2DM) [24, 25]. Elevated levels of these proteins in IBD/IBS are in line with previous studies, showing that insulin dysregulation plays a role in colon inflammation [26], and that IBD, CRC and T2DM are commonly occurring interrelated clinical problems, sharing a common basis influenced by inflammatory process, metabolic perturbations and microbiota dysbiosis [27]. Although several reports suggested some of these proteins (i.e. AXIN1, CASP-8, ST1 A1, and TNFSF14) could be utilized to distinguish UC from IBS [9, 28], our study did not replicate these findings, potentially due to the treatment naivety of the cohort.

Our report also identified several proteins with decreased levels in diseased individuals, which further discerned healthy and IBD/IBS groups. The most pronounced difference was identified for ROR1 (receptor tyrosine kinase-like orphan receptor 1), as well as for CCDC80 (coiled-coil domain-containing 80) and ACP6 (lysophospatidic acid phosphatase type 6). The latter two are linked to obesity and related metabolic diseases [29, 30]. The levels of IL-10 (interleukin-10) were also decreased in all the disease groups. IL-10, usually considered the most important anti-inflammatory cytokine, was significantly depleted in the IBS compared to the healthy group, in line with previous studies [31]. Reduced levels of IL-10 in the CD and UC, however, were not statistically significant.

Distinct inflammatory status of IBD and IBS was reflected in different levels of CRP and several other proteins, with the highest selectivity and specificity identified for IL-17 A (interleukin-17 A). The pro-inflammatory role of T_H_17 cells and IL-17 A in IBD is well-documented [32], with recent studies suggesting that disturbed microbiota metabolism drives T_H_17 activation and colitis [33]. Our study reports higher levels of IL-17 A in IBD confirming previously published results [34] and showing that this effect is also present early in the disease progression. IL-17 A was markedly increased in UC patients, well differentiating UC and IBS, as well as UC and healthy groups. We detected no changes in IL-17 A levels between the healthy and IBS groups, which is in line with previously reported findings [7].

Several proteins discerned IBD from IBS due to their significantly lower levels in IBS, compared to both IBD and healthy individuals. Most notable were TNFRSF9 (tumour necrosis factor receptor superfamily member 9) and CXCL10 (C-X-C motif chemokine 10), both associated with chronic inflammation [35] and IBD [36]. These proteins differentiated CD from IBS, but surprisingly their levels were not elevated in CD compared to the heathy group. Another protein distinguishing IBD from IBS was TYMP (thymidine phosphorylase), a pyrimidine-metabolizing enzyme implicated in several inflammatory diseases, as well as in IBD [37]. TYMP was increased in CD compared to IBS and healthy groups. In addition, unlike all the other proteins in this study, TYMP differentiated well between CD and UC, although the large effect size was not supported by statistical significance.

Although CRP levels were statistically different between IBD and IBS and CD and IBS groups, its predictive power based on ROC was lower, not meeting the set criteria. Nevertheless, CRP correlated mostly with IL-17 A and TNFRSF9 (Fig. 4), indicating these markers reflect changes in inflammatory status of IBD and IBS. The level of correlation and distinct values between diagnoses still warrant IL-17 A and TNFRSF9 as interesting potential biomarkers additional to CRP.

The protein with a completely distinctive pattern in this study was GAL (galanin). A neurotransmitter widely distributed in the enteric nerve terminals lining the GI tract; GAL is involved in regulation of gastrointestinal motility, smooth muscle contractility and fluid secretion [38]. It is also implicated in glucose metabolism, alleviating insulin resistance and lowering the possibility of developing T2DM [39], as well as in the inflammatory response in the gut [40], improving disease outcome in animal colitis models [41]. We identified decreased GAL levels in both CD and UC compared to healthy and IBS groups, with significant differences evident between H and IBD, as well as between IBD and IBS groups. Consequently, our findings suggest regulatory implications of GAL in metabolic and inflammatory disease pathways and its potential role as an IBD biomarker.

*Enterobacteriaceae* was the only bacterial family associated with proteins significantly elevated in the disease groups. Increased abundance and expansion of *Enterobacteriaceae* is implicated in both IBD and IBS [13, 42, 43], and has been linked to increased pro-inflammatory cytokine production and intestinal inflammation [44]. On the contrary, *Ruminococcaceae*, *Verrucomicrobiaceae* and *Christensenellaceae* clustered with proteins found decreased in disease groups. These bacterial families contain species with beneficial effects on intestinal homeostasis and are often found depleted in IBD patients. Anti-inflammatory properties of *Ruminococcaceae* family, *Faecalibacterium prausnitzii* in particular, are well-documented [45] and include the ability to modulate mucosal immune responses of the host through the production of short-chain fatty acids (SCFAs). Favourable effects of SCFA include inhibition of pro-inflammatory cytokine expression, maintenance of tight junction integrity and production of antimicrobial peptides. Depletion of SCFA-producing bacterial species could therefore contribute to mucosal inflammation. Favourable effects on intestinal homeostasis are also documented for *Akkermansia muciniphila*, the main intestinal representative of *Verrucomicrobiaceae* family, important for the maintenance of mucus layer integrity [46]. In our study, *A. muciniphila* accounted for all identified *Verrucomicrobiaceae* OTUs. *Pasteurellaceae* associated with proteins differentiating IBD and IBS, displaying higher abundance in the faeces of UC patients compared to both healthy and IBS groups. The increased levels of *Haemophilus parainfluenzae*, which accounted for all *Pasteurellaceae* OTUs identified in this study, have been documented in patients with UC and associated with raised quantities of acylcarnitine, serving as a potential faecal biomarker for IBD [47, 48].

Based on the capacity of each biomarker to be used as a predictor of a diagnosis, reported as the area under the ROC curve (AUC), serum proteins displayed higher AUC for each comparison as opposed to faecal bacterial families. This may, at least in part, be attributed to high intrinsic interindividual variability of faecal microbiota composition. The sole exception was the UC vs CD comparison, where predictive power is overall lower, but the dominant prognostic marker is the abundance of *Turicibacteraceae* in faeces.

**Fig. 5:**
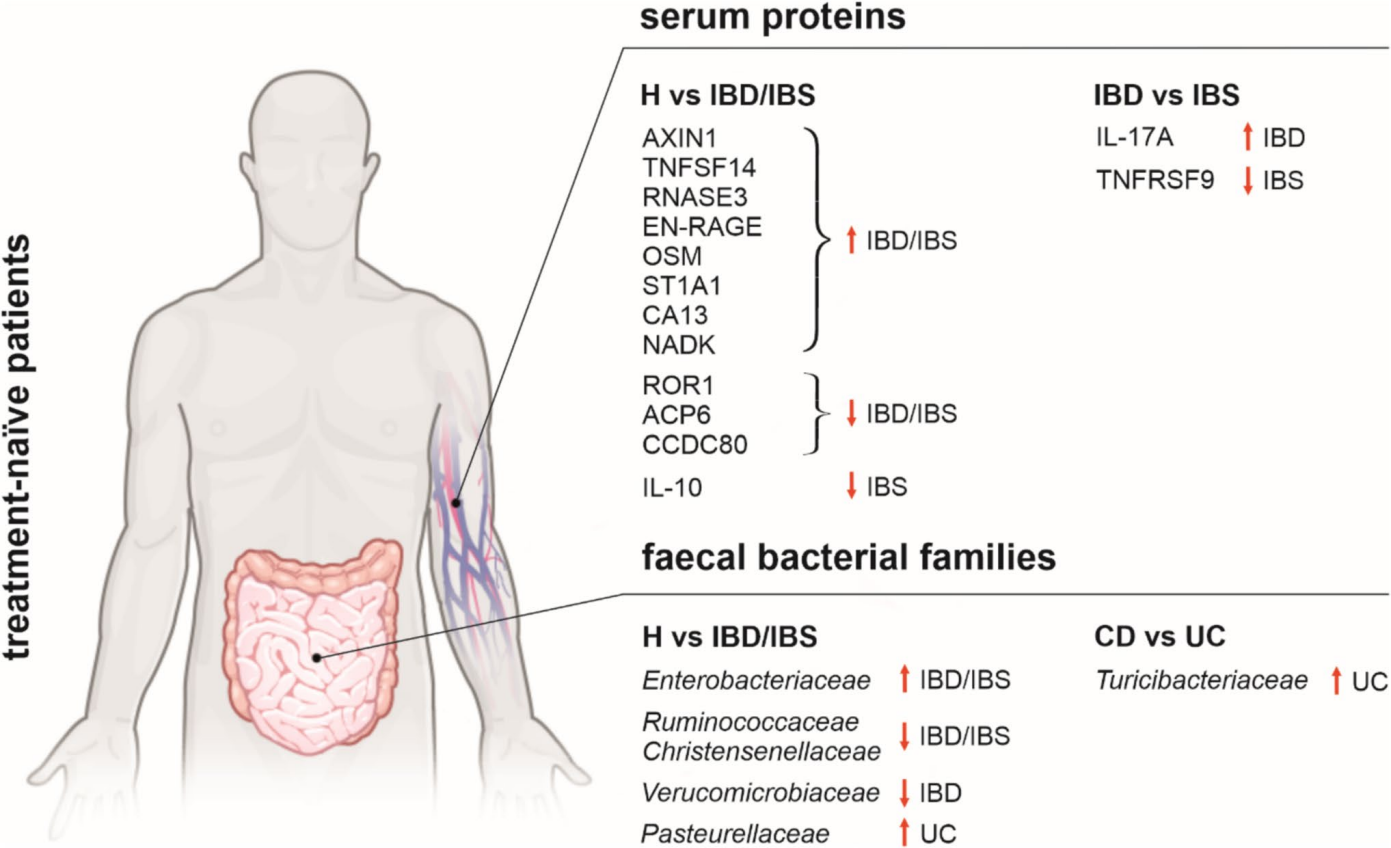
Markers with highest specificity/sensitivity and predictivity for discriminating study groups based on ROC analysis

There are several potential limitations to this work. A relatively small cohort can impact sensitivity to subtle effects, especially when profiling faecal microbiota composition where interindividual differences are inherently high [49]. Additionally, the PEA method provides only relative quantification of serum proteins so the results cannot be directly compared to methods measuring absolute protein levels (i.e. ELISA). Furthermore, several markers of potential interest (IFN-γ, TNF-α, IL-1 α and IL-13) were excluded from the analysis because they were not detectable in > 40% of samples. Finally, this study does not include a validation cohort to confirm the findings. Despite these constraints, our study covers several gastrointestinal conditions, and it enables comparison between CD, UC and IBS patients during the rarely investigated treatment-naïve stage, including also healthy individuals, thus providing valuable and unique insight into disease onset. The findings revealed characteristic disease signatures in both inflammatory and metabolism-related serum proteins with potential for discerning IBD and IBS patients from healthy individuals, as well as between IBD and IBS (Fig. 5).

In addition, the changes in protein levels were associated with faecal microbiota content, contributing to better characterization of the complex interplay at the host-microbiota interface, improved understanding of pathogenic mechanisms of gastrointestinal disorders, and identification of potential novel non-invasive diagnostic markers of IBD and IBS.

## Supplementary Information

Below is the link to the electronic supplementary material.

Supplementary file1 (DOCX 114 KB)

## Data Availability

The underlying data are available in the article and in its online supplementary material. In addition, the raw dataset used in this study is available from the corresponding author upon reasonable request.
