# Supplementary material for: Serum proteins and faecal microbiota as potential biomarkers in newly diagnosed, treatment-naïve inflammatory bowel disease and irritable bowel syndrome patients
Source: J Mol Med (Berl). 2025 Jun 7;103(8):963–74. doi: 10.1007/s00109-025-02558-5 (PMC12343743; doi:10.1007/s00109-025-02558-5)
Supplement: Supplementary file 1 — Supplementary file1 (DOCX 114 KB) [file 109_2025_2558_MOESM1_ESM.docx]

**Serum proteins and faecal microbiota as potential biomarkers in newly diagnosed, treatment-naïve inflammatory bowel disease and irritable bowel syndrome patients**

**Journal of Molecular Medicine**

Mario Matijašić, Anja Barešić, Hana Čipčić Paljetak^*^, Mihaela Perić, Marina Panek, Ana Kunović, Dina Ljubas Kelečić, Darija Vranešić Bender, Katja Grubelić Ravić, Dunja Rogić, Margareta Antolic, Ivana Horvat, Ivana Kraljević, Marko Banić, Željko Krznarić, Donatella Verbanac

* Corresponding author: Hana Čipčić Paljetak, +385 14590069, email: [hana.paljetak@mef.hr](mailto:hana.paljetak@mef.hr)

Center for Translational and Clinical Research, University of Zagreb School of Medicine, Zagreb, Croatia

Supplementary Table S1 List of all proteins in OLINK inflammation panel

| Full name | Biomarker | UniProt No | % below LOD |
| --- | --- | --- | --- |
| Adenosine Deaminase | ADA | P00813 | 0.00 |
| Artemin | ARTN | Q5T4W7 | 72.58 |
| Axin-1 | AXIN1 | O15169 | 8.06 |
| Beta-nerve growth factor | β-NGF | P01138 | 0.00 |
| Caspase-8 | CASP-8 | Q14790 | 0.00 |
| C-C motif chemokine 3 | CCL3 | P10147 | 0.00 |
| C-C motif chemokine 4 | CCL4 | P13236 | 0.00 |
| C-C motif chemokine 19 | CCL19 | Q99731 | 0.00 |
| C-C motif chemokine 20 | CCL20 | P78556 | 0.00 |
| C-C motif chemokine 23 | CCL23 | P55773 | 0.00 |
| C-C motif chemokine 25 | CCL25 | O15444 | 0.00 |
| C-C motif chemokine 28 | CCL28 | Q9NRJ3 | 1.61 |
| CD40L receptor | CD40 | P25942 | 0.00 |
| CUB domain-containing protein 1 | CDCP1 | Q9H5V8 | 0.00 |
| C-X-C motif chemokine 1 | CXCL1 | P09341 | 0.00 |
| C-X-C motif chemokine 5 | CXCL5 | P42830 | 0.00 |
| C-X-C motif chemokine 6 | CXCL6 | P80162 | 0.00 |
| C-X-C motif chemokine 9 | CXCL9 | Q07325 | 0.00 |
| C-X-C motif chemokine 10 | CXCL10 | P02778 | 0.00 |
| C-X-C motif chemokine 11 | CXCL11 | O14625 | 0.00 |
| Cystatin D | CST5 | P28325 | 0.00 |
| Delta and Notch-like epidermal growth factor-related receptor | DNER | Q8NFT8 | 0.00 |
| Eotaxin | CCL11 | P51671 | 0.00 |
| Eukaryotic translation initiation factor 4E-binding protein 1 | 4E-BP1 | Q13541 | 0.00 |
| Fibroblast growth factor 5 | FGF-5 | P12034 | 6.45 |
| Fibroblast growth factor 19 | FGF-19 | O95750 | 0.00 |
| Fibroblast growth factor 21 | FGF21 | Q9NSA1 | 1.61 |
| Fibroblast growth factor 23 | FGF-23 | Q9GZV9 | 6.45 |
| Fms-related tyrosine kinase 3 ligand | Flt3L | P49771 | 0.00 |
| Fractalkine | CX3CL1 | P78423 | 0.00 |
| Glial cell line-derived neurotrophic factor | GDNF | P39905 | 3.23 |
| Hepatocyte growth factor | HGF | P14210 | 0.00 |
| Interferon gamma | IFN-γ | P01579 | 77.42 |
| Interleukin-1 alpha | IL-1α | P01583 | 98.39 |
| Interleukin-2 | IL-2 | P60568 | 83.87 |
| Interleukin-2 receptor subunit beta | IL-2RB | P14784 | 72.58 |
| Interleukin-4 | IL-4 | P05112 | 83.87 |
| Interleukin-5 | IL-5 | P05113 | 46.77 |
| Interleukin-6 | IL-6 | P05231 | 25.81 |
| Interleukin-7 | IL-7 | P13232 | 0.00 |
| Interleukin-8 | IL-8 | P10145 | 0.00 |
| Interleukin-10 | IL-10 | P22301 | 0.00 |
| Interleukin-10 receptor subunit alpha | IL-10RA | Q13651 | 40.32 |
| Interleukin-10 receptor subunit beta | IL-10RB | Q08334 | 0.00 |
| Interleukin-12 subunit beta | IL-12B | P29460 | 0.00 |
| Interleukin-13 | IL-13 | P35225 | 66.13 |
| Interleukin-15 receptor subunit alpha | IL-15RA | Q13261 | 22.58 |
| Interleukin-17A | IL-17A | Q16552 | 30.65 |
| Interleukin-17C | IL-17C | Q9P0M4 | 14.52 |
| Interleukin-18 | IL-18 | Q14116 | 0.00 |
| Interleukin-18 receptor 1 | IL-18R1 | Q13478 | 0.00 |
| Interleukin-20 | IL-20 | Q9NYY1 | 67.74 |
| Interleukin-20 receptor subunit alpha | IL-20RA | Q9UHF4 | 85.48 |
| Interleukin-22 receptor subunit alpha-1 | IL-22 RA1 | Q8N6P7 | 100.00 |
| Interleukin-24 | IL-24 | Q13007 | 70.97 |
| Interleukin-33 | IL-33 | O95760 | 96.77 |
| Latency-associated peptide transforming growth factor beta-1 | LAP TGF-β-1 | P01137 | 0.00 |
| Leukemia inhibitory factor | LIF | P15018 | 67.74 |
| Leukemia inhibitory factor receptor | LIF-R | P42702 | 0.00 |
| Macrophage colony-stimulating factor 1 | CSF-1 | P09603 | 0.00 |
| Matrix metalloproteinase-1 | MMP-1 | P03956 | 0.00 |
| Matrix metalloproteinase-10 | MMP-10 | P09238 | 0.00 |
| Monocyte chemotactic protein 1 | MCP-1 | P13500 | 0.00 |
| Monocyte chemotactic protein 2 | MCP-2 | P80075 | 0.00 |
| Monocyte chemotactic protein 3 | MCP-3 | P80098 | 1.61 |
| Monocyte chemotactic protein 4 | MCP-4 | Q99616 | 0.00 |
| Natural killer cell receptor 2B4 | CD244 | Q9BZW8 | 0.00 |
| Neurotrophin-3 | NT-3 | P20783 | 1.61 |
| Neurturin | NRTN | Q99748 | 56.45 |
| Oncostatin-M | OSM | P13725 | 0.00 |
| Osteoprotegerin | OPG | O00300 | 0.00 |
| Programmed cell death 1 ligand 1 | PD-L1 | Q9NZQ7 | 0.00 |
| Protein S100-A12 | EN-RAGE | P80511 | 0.00 |
| Signaling lymphocytic activation molecule | SLAMF1 | Q13291 | 0.00 |
| SIR2-like protein 2 | SIRT2 | Q8IXJ6 | 16.13 |
| STAM-binding protein | STAMBP | O95630 | 0.00 |
| Stem cell factor | SCF | P21583 | 0.00 |
| Sulfotransferase 1A1 | ST1A1 | P50225 | 1.61 |
| T cell surface glycoprotein CD6 isoform | CD6 | P30203 | 0.00 |
| T-cell surface glycoprotein CD5 | CD5 | P06127 | 0.00 |
| T-cell surface glycoprotein CD8 alpha chain | CD8A | P01732 | 0.00 |
| Thymic stromal lymphopoietin | TSLP | Q969D9 | 93.55 |
| TNF-beta | TNFB | P01374 | 0.00 |
| TNF-related activation-induced cytokine | TRANCE | O14788 | 0.00 |
| TNF-related apoptosis-inducing ligand | TRAIL | P50591 | 0.00 |
| Transforming growth factor alpha | TGF-α | P01135 | 0.00 |
| Tumor necrosis factor (Ligand) superfamily. member 12 | TWEAK | O43508 | 0.00 |
| Tumor necrosis factor | TNF | P01375 | 69.35 |
| Tumor necrosis factor ligand superfamily member 14 | TNFSF14 | O43557 | 0.00 |
| Tumor necrosis factor receptor superfamily member 9 | TNFRSF9 | Q07011 | 0.00 |
| Urokinase-type plasminogen activator | uPA | P00749 | 0.00 |
| Vascular endothelial growth factor A | VEGF-A | P15692 | 0.00 |

Supplementary Table S2 List of all proteins in OLINK metabolism panel

| Full name | Biomarker | UniProt No | % below LOD |
| --- | --- | --- | --- |
| Adenosylhomocysteinase | AHCY | P23526 | 100.00 |
| Adhesion G protein-coupled receptor E2 | ADGRE2 | Q9UHX3 | 0.00 |
| Adhesion G-protein coupled receptor G2 | ADGRG2 | Q8IZP9 | 0.00 |
| Amyloid-like protein 1 | APLP1 | P51693 | 0.00 |
| Angiopoietin-2 | ANGPT2 | O15123 | 0.00 |
| Angiopoietin-related protein 1 | ANGPTL1 | O95841 | 0.00 |
| Angiopoietin-related protein 7 | ANGPTL7 | O43827 | 0.00 |
| Annexin A4 | ANXA4 | P09525 | 98.39 |
| Annexin A11 | ANXA11 | P50995 | 77.42 |
| Appetite-regulating hormone | GHRL | Q9UBU3 | 0.00 |
| Arginase-1 | ARG1 | P05089 | 16.13 |
| Aromatic-L-amino-acid decarboxylase | DDC | P20711 | 0.00 |
| B-cell antigen receptor complex-associated protein beta chain | CD79B | P40259 | 0.00 |
| Cadherin-2 | CDH2 | P19022 | 0.00 |
| Cadherin-related family member 5 | CDHR5 | Q9HBB8 | 0.00 |
| Calsyntenin-2 | CLSTN2 | Q9H4D0 | 0.00 |
| Carbonic anhydrase 13 | CA13 | Q8N1Q1 | 8.06 |
| Catechol O-methyltransferase | COMT | P21964 | 77.42 |
| Cathepsin O | CTSO | P43234 | 0.00 |
| CD2-associated protein | CD2AP | Q9Y5K6 | 0.00 |
| Chordin-like protein 2 | CHRDL2 | Q6WN34 | 0.00 |
| Clusterin-like protein 1 | CLUL1 | Q15846 | 0.00 |
| Coiled-coil domain-containing protein 80 | CCDC80 | Q76M96 | 0.00 |
| Crk-like protein | CRKL | P46109 | 54.84 |
| C-type lectin domain family 5 member A | CLEC5A | Q9NY25 | 0.00 |
| CXADR-like membrane protein | CLMP | Q9H6B4 | 0.00 |
| Diablo homolog. mitochondrial | DIABLO | Q9NR28 | 100.00 |
| Dihydropteridine reductase | QDPR | P09417 | 0.00 |
| Dipeptidyl peptidase 2 | DPP7 | Q9UHL4 | 1.61 |
| Disabled homolog 2 | DAB2 | P98082 | 100.00 |
| DNA-(apurinic or apyrimidinic site) lyase | APEX1 | P27695 | 29.03 |
| Ectonucleoside triphosphate diphosphohydrolase 5 | ENTPD5 | O75356 | 0.00 |
| Ectonucleotide pyrophosphatase/phosphodiesterase family member 7 | ENPP7 | Q6UWV6 | 0.00 |
| Eosinophil cationic protein | RNASE3 | P12724 | 0.00 |
| Fc receptor-like protein 1 | FCRL1 | Q96LA6 | 0.00 |
| Fructose-1.6-bisphosphatase 1 | FBP1 | P09467 | 62.90 |
| Galanin peptides | GAL | P22466 | 0.00 |
| Gamma-enolase | ENO2 | P09104 | 0.00 |
| Glutaredoxin-1 | GLRX | P35754 | 51.61 |
| GRB2-related adapter protein 2 | GRAP2 | O75791 | 12.90 |
| Hepatoma-derived growth factor | HDGF | P51858 | 61.29 |
| Inactive tyrosine-protein kinase transmembrane receptor ROR1 | ROR1 | Q01973 | 0.00 |
| Insulin-like growth factor-binding protein-like 1 | IGFBPL1 | Q8WX77 | 0.00 |
| Integrin beta-7 | ITGB7 | P26010 | 53.23 |
| Kallikrein-10 | KLK10 | O43240 | 0.00 |
| Kynurenine-oxoglutarate transaminase 1 | KYAT1 | Q16773 | 0.00 |
| Large proline-rich protein BAG6 | BAG6 | P46379 | 0.00 |
| Leucine-rich repeats and immunoglobulin-like domains protein 1 | LRIG1 | Q96JA1 | 0.00 |
| Leukocyte immunoglobulin-like receptor subfamily A member 5 | LILRA5 | A6NI73 | 0.00 |
| Low-density lipoprotein receptor-related protein 11 | LRP11 | Q86VZ4 | 0.00 |
| Lysophosphatidic acid phosphatase type 6 | ACP6 | Q9NPH0 | 0.00 |
| Meprin A subunit beta | MEP1B | Q16820 | 20.97 |
| Meteorin-like protein | METRNL | Q641Q3 | 0.00 |
| Multiple coagulation factor deficiency protein 2 | MCFD2 | Q8NI22 | 0.00 |
| NAD kinase | NADK | O95544 | 0.00 |
| Nectin-2 | NECTIN2 | Q92692 | 0.00 |
| Neural proliferation differentiation and control protein 1 | NPDC1 | Q9NQX5 | 0.00 |
| Neuronal pentraxin receptor | NPTXR | O95502 | 0.00 |
| Nodal modulator 1 | NOMO1 | Q15155 | 0.00 |
| N-terminal prohormone brain natriuretic peptide | NT-proBNP | NA | 14.52 |
| Paired immunoglobulin-like type 2 receptor beta | PILRB | Q9UKJ0 | 0.00 |
| Peptidyl-prolyl cis-trans isomerase FKBP4 | FKBP4 | Q02790 | 87.10 |
| Phosphoprotein with glycosphingolipid-enriched microdomains 1 | PAG1 | Q9NWQ8 | 11.29 |
| Pro-cathepsin H | CTSH | P09668 | 20.97 |
| Protein FAM3C | FAM3C | Q92520 | 0.00 |
| Protein phosphatase inhibitor 2 | PPP1R2 | P41236 | 6.45 |
| Protein S100-P | S100P | P25815 | 90.32 |
| Regenerating islet-derived protein 4 | REG4 | Q9BYZ8 | 0.00 |
| Reticulon-4 receptor | RTN4R | Q9BZR6 | 0.00 |
| Retinal dehydrogenase 1 | ALDH1A1 | P00352 | 1.61 |
| Ribosyldihydronicotinamide dehydrogenase [quinone] | NQO2 | P16083 | 100.00 |
| Scavenger receptor cysteine-rich domain-containing group B protein | SSC4D | Q8WTU2 | 22.58 |
| Sclerostin | SOST | Q9BQB4 | 0.00 |
| Semaphorin-3F | SEMA3F | Q13275 | 0.00 |
| Serpin B6 | SERPINB6 | P35237 | 0.00 |
| Serpin B8 | SERPINB8 | P50452 | 0.00 |
| Sialic acid-binding Ig-like lectin 7 | SIGLEC7 | Q9Y286 | 0.00 |
| Sialomucin core protein 24 | CD164 | Q04900 | 0.00 |
| Soluble calcium-activated nucleotidase 1 | CANT1 | Q8WVQ1 | 0.00 |
| Sulfatase-modifying factor 2 | SUMF2 | Q8NBJ7 | 0.00 |
| Synaptosomal-associated protein 23 | SNAP23 | O00161 | 38.71 |
| Syndecan-4 | SDC4 | P31431 | 0.00 |
| T-cell surface glycoprotein CD1c | CD1C | P29017 | 0.00 |
| Thimet oligopeptidase | THOP1 | P52888 | 0.00 |
| Thioredoxin domain-containing protein 5 | TXNDC5 | Q8NBS9 | 1.61 |
| Thymidine phosphorylase | TYMP | P19971 | 0.00 |
| Thyrotropin subunit beta | TSHB | P01222 | 0.00 |
| Trefoil factor 2 | TFF2 | Q03403 | 0.00 |
| Tubulointerstitial nephritis antigen-like | TINAGL1 | Q9GZM7 | 0.00 |
| Tyrosine-protein kinase receptor TYRO3 | TYRO3 | Q06418 | 0.00 |
| Ubiquitin carboxyl-terminal hydrolase 8 | USP8 | P40818 | 64.52 |
| Versican core protein | VCAN | P13611 | 0.00 |

Supplementary Table S3 Differentially expressed serum protein biomarkers between groups (H – healthy, CD – Crohn’s disease, UC – ulcerative colitis, IBS – irritable bowel syndrome, IBD – inflammatory bowel disease). Statistical significance of Wilcoxon paired test is given as FDR-corrected p-value (*<0,05, **<0,01, ***<0,001, ****<0,0001). Median ± SD values for these proteins are given in Supplemental Table S4

|  |  | **H vs CD** | **H vs UC** | **H vs IBS** | **UC vs CD** | **UC vs IBS** | **CD vs IBS** | **H vs IBD** | **IBD vs IBS** |
| --- | --- | --- | --- | --- | --- | --- | --- | --- | --- |
| inflammation panel | AXIN1 | ** | ** | *** |  |  |  | *** |  |
|  | CASP-8 | * | * |  |  |  |  | *** |  |
|  | CCL11 |  |  |  |  |  |  |  | * |
|  | CCL19 |  |  |  |  |  |  |  | * |
|  | CCL20 | * | * |  |  | * | * | ** | ** |
|  | CCL28 |  |  | * |  | * | * |  | *** |
|  | CD5 |  |  |  |  |  |  |  | * |
|  | CD6 |  |  |  |  |  |  |  | * |
|  | CDCP1 |  |  |  |  |  |  |  | * |
|  | CXCL10 |  |  | * |  |  | ** |  | *** |
|  | CXCL11 |  |  |  |  |  | * |  | * |
|  | CXCL9 |  |  |  |  | ** | *** |  | **** |
|  | EN-RAGE | ** | ** | ** |  |  |  | **** |  |
|  | FGF-19 | * |  | * |  |  |  | * |  |
|  | FGF-23 |  |  |  |  |  | * |  | * |
|  | HGF | * |  |  |  |  |  | * |  |
|  | IL-17A |  | * |  |  | *** | * | * | **** |
|  | IL-10 |  |  | *** |  | * |  |  | * |
|  | IL-18 |  |  |  |  |  | * |  | * |
|  | IL-6 |  |  | * |  |  | * |  |  |
|  | LIF-R |  |  |  |  |  |  |  | * |
|  | OPG |  |  | * |  |  |  |  |  |
|  | OSM | **** | ** | ** |  |  | * | **** |  |
|  | PD-L1 |  |  |  |  |  |  |  |  |
|  | SIRT-2 | * | * | * |  |  |  | * |  |
|  | ST1A1 | *** | ** | *** |  |  |  | **** |  |
|  | TGF-α | ** |  |  |  |  |  | ** |  |
|  | TNFRSF9 |  |  | * |  | ** | ** |  | **** |
|  | TNFSF14 | **** | *** | **** |  |  |  | **** |  |
|  | VEGFA | * |  |  |  |  | * | * | * |
| metabolism panel | ACP6 | * | * | * |  |  |  | ** |  |
|  | ADGRE2 |  |  | ** |  |  |  | * |  |
|  | ADGRG2 | ** |  |  |  |  |  | ** |  |
|  | ANGPT2 |  |  |  |  |  |  |  | * |
|  | APEX1 | * | * | * |  |  |  | ** |  |
|  | CA13 | * | *** | *** |  |  |  | *** |  |
|  | CCDC80 |  | * | ** |  |  |  | ** |  |
|  | CD164 |  | * |  |  |  |  | ** |  |
|  | CD79B |  |  |  |  |  | * |  | * |
|  | CHRDL2 |  |  | * |  |  |  |  | * |
|  | CLEC5A | * | * |  |  |  |  | ** |  |
|  | DPP7 | * | * | * |  |  |  | ** |  |
|  | FAM3C |  |  | * |  |  |  |  |  |
|  | GAL | * | * |  |  | * | * | ** | ** |
|  | GRAP2 | * | * |  |  |  |  | ** |  |
|  | LILRA5 | * |  |  |  |  |  | ** |  |
|  | NADK | ** | *** | *** |  |  |  | **** |  |
|  | PILRB |  |  |  |  |  | * |  | ** |
|  | PPP1R2 |  |  |  |  |  |  | ** |  |
|  | RNASE3 | *** | *** | *** |  |  | * | **** |  |
|  | ROR1 | ** | * | ** |  |  |  | *** |  |
|  | SIGLEC7 |  |  | * |  |  | * |  | * |
|  | SNAP23 | ** | ** | ** |  |  |  | *** |  |
|  | SOST |  |  | * |  | * | * |  | ** |
|  | TYMP | * |  |  |  |  | *** | * | ** |

Supplementary Table S4 Median values of serum protein biomarkers and their standard deviations (H – healthy, CD – Crohn’s disease, UC – ulcerative colitis, IBS – irritable bowel syndrome)

|  |  | H | | CD | | UC | | IBS | |
| --- | --- | --- | --- | --- | --- | --- | --- | --- | --- |
|  |  | median | SD | median | SD | median | SD | median | SD |
| inflammation panel | AXIN1 | 0.8650 | 0.2923 | 1.9349 | 0.8213 | 1.7436 | 0.5783 | 1.7654 | 0.6102 |
|  | CASP-8 | 2.1431 | 0.2808 | 2.5191 | 0.4538 | 2.4394 | 0.3883 | 2.3284 | 0.3530 |
|  | CCL11 | 7.9359 | 0.4088 | 7.8358 | 0.7159 | 8.0889 | 0.2991 | 7.6634 | 0.3743 |
|  | CCL19 | 9.2282 | 0.8302 | 10.0043 | 0.8430 | 9.5505 | 1.0900 | 9.3087 | 0.4353 |
|  | CCL20 | 4.6134 | 1.3362 | 5.5211 | 1.2013 | 5.6064 | 1.7283 | 5.2379 | 0.8041 |
|  | CCL28 | 2.2629 | 0.7951 | 2.2416 | 0.4777 | 2.1678 | 0.2765 | 1.8748 | 0.2322 |
|  | CD5 | 4.3405 | 0.4772 | 4.6100 | 0.4793 | 4.5170 | 0.3347 | 4.3234 | 0.2966 |
|  | CD6 | 5.6351 | 0.7427 | 5.4931 | 0.5604 | 5.2797 | 0.4500 | 5.1938 | 0.3841 |
|  | CDCP1 | 2.6032 | 0.4924 | 3.1749 | 0.8345 | 2.8078 | 0.3947 | 2.5068 | 0.5637 |
|  | CXCL10 | 10.0438 | 0.9478 | 10.0848 | 0.9849 | 9.3359 | 0.8511 | 9.1362 | 0.5062 |
|  | CXCL11 | 8.1024 | 0.6709 | 8.7710 | 0.9172 | 8.1399 | 1.7002 | 8.0003 | 0.6965 |
|  | CXCL9 | 7.4359 | 0.6924 | 7.9494 | 1.4552 | 7.5830 | 1.3938 | 6.6876 | 0.5216 |
|  | EN-RAGE | 3.7351 | 0.8022 | 5.0646 | 1.0206 | 4.7091 | 0.6719 | 5.1294 | 0.9099 |
|  | FGF-19 | 8.2025 | 0.6799 | 7.3742 | 0.6455 | 7.9800 | 0.8701 | 7.7619 | 0.8257 |
|  | FGF-23 | 1.6856 | 0.3741 | 1.9821 | 0.7199 | 1.5258 | 1.0739 | 1.5009 | 0.2740 |
|  | HGF | 9.5394 | 0.3482 | 10.1190 | 0.4942 | 9.8204 | 0.6312 | 9.6358 | 0.6079 |
|  | IL-17A | 1.0518 | 1.1491 | 1.5910 | 1.2191 | 1.7268 | 0.4465 | 1.1594 | 0.1376 |
|  | IL-10 | 3.9233 | 0.4502 | 3.1904 | 0.8432 | 3.5703 | 0.5415 | 3.0898 | 0.4663 |
|  | IL-18 | 8.5657 | 0.5399 | 8.6042 | 0.6569 | 8.1406 | 0.7837 | 8.2156 | 0.5390 |
|  | IL-6 | 3.4093 | 0.4211 | 3.7960 | 1.1913 | 3.1448 | 0.6315 | 2.7461 | 0.5313 |
|  | LIF-R | 3.0234 | 0.3117 | 3.1753 | 0.2265 | 3.1312 | 0.2979 | 3.0215 | 0.2417 |
|  | OPG | 10.3997 | 0.3533 | 10.2903 | 0.2877 | 9.9866 | 0.4478 | 10.1070 | 0.3366 |
|  | OSM | 6.6369 | 0.4802 | 8.2308 | 0.6281 | 7.7525 | 0.9761 | 7.5179 | 0.8599 |
|  | PD-L1 | 5.2976 | 0.3457 | 5.2950 | 0.4196 | 4.9482 | 0.3361 | 5.0393 | 0.2967 |
|  | SIRT-2 | 2.1031 | 0.4164 | 2.8580 | 0.8892 | 2.9769 | 0.7147 | 2.7094 | 0.7269 |
|  | ST1A1 | 1.1391 | 0.5094 | 3.4407 | 1.1548 | 2.3719 | 0.9686 | 3.2515 | 1.4053 |
|  | TGF-α | 5.4677 | 0.4906 | 6.6213 | 0.5202 | 5.9881 | 0.8080 | 6.2304 | 0.6235 |
|  | TNFRSF9 | 6.8114 | 0.4280 | 6.8913 | 0.6777 | 6.8736 | 0.3383 | 6.4335 | 0.3603 |
|  | TNFSF14 | 4.9645 | 0.6028 | 6.8002 | 0.7152 | 6.5279 | 1.0377 | 6.4450 | 0.7515 |
|  | VEGFA | 10.5704 | 0.4624 | 10.9038 | 0.6177 | 10.6843 | 0.4801 | 10.4566 | 0.5447 |
| metabolism panel | ACP6 | 3.4311 | 0.5788 | 2.7620 | 0.4574 | 2.9190 | 0.5720 | 2.8706 | 0.4049 |
|  | ADGRE2 | 3.4224 | 0.2452 | 3.0545 | 0.5212 | 3.0873 | 0.5210 | 3.0733 | 0.3466 |
|  | ADGRG2 | 1.9664 | 0.2539 | 1.5207 | 0.2161 | 1.6830 | 0.4364 | 1.7136 | 0.3820 |
|  | ANGPT2 | 1.7371 | 0.4645 | 2.1195 | 0.3759 | 2.2023 | 0.6638 | 1.7699 | 0.4174 |
|  | APEX1 | 0.0739 | 0.1794 | 0.8069 | 1.1086 | 0.4980 | 0.4121 | 0.5521 | 0.6630 |
|  | CA13 | 1.5198 | 0.5906 | 3.3896 | 1.3886 | 2.9812 | 0.9455 | 2.9587 | 1.2764 |
|  | CCDC80 | 5.2636 | 0.3816 | 4.8723 | 0.5008 | 4.7994 | 0.4244 | 4.7847 | 0.3980 |
|  | CD164 | 4.8939 | 0.3806 | 5.1819 | 0.4776 | 5.2040 | 0.3095 | 5.0486 | 0.3759 |
|  | CD79B | 1.7092 | 0.4158 | 2.0919 | 0.2998 | 1.8090 | 0.3011 | 1.7359 | 0.3076 |
|  | CHRDL2 | 3.4869 | 0.5688 | 3.4580 | 0.7046 | 3.1592 | 0.6947 | 2.9250 | 0.6167 |
|  | CLEC5A | 3.8862 | 0.2271 | 4.2123 | 0.4980 | 4.1907 | 0.3338 | 4.0517 | 0.4387 |
|  | DPP7 | 0.6603 | 0.4201 | 1.3370 | 0.5739 | 1.0668 | 0.3194 | 1.2858 | 0.3338 |
|  | FAM3C | 6.5122 | 0.3735 | 6.2825 | 0.3648 | 6.1434 | 0.2849 | 6.1778 | 0.3173 |
|  | GAL | 6.1201 | 0.8788 | 5.2676 | 0.7880 | 5.1596 | 0.5711 | 5.8621 | 0.8462 |
|  | GRAP2 | 0.0499 | 0.2111 | 0.3336 | 0.5283 | 0.3470 | 0.2396 | 0.2616 | 0.3804 |
|  | LILRA5 | 3.2865 | 0.3261 | 3.7366 | 0.5267 | 3.4518 | 0.5305 | 3.4545 | 0.4533 |
|  | NADK | 3.0848 | 0.5677 | 4.2302 | 1.3576 | 4.3995 | 0.5303 | 4.6112 | 0.8987 |
|  | PILRB | 5.2153 | 0.6732 | 5.8010 | 0.7603 | 5.5045 | 0.4777 | 5.1099 | 0.6908 |
|  | PPP1R2 | 0.8740 | 0.3341 | 1.2489 | 0.5480 | 1.2423 | 0.1493 | 1.0963 | 0.3997 |
|  | RNASE3 | 4.4319 | 1.1504 | 7.1753 | 0.8149 | 6.6091 | 0.8887 | 6.3305 | 1.1947 |
|  | ROR1 | 2.6574 | 0.3147 | 2.0922 | 0.3377 | 2.1710 | 0.2672 | 2.1604 | 0.4109 |
|  | SIGLEC7 | 3.0541 | 0.4105 | 2.8951 | 0.3218 | 2.7274 | 0.6400 | 2.5330 | 0.3274 |
|  | SNAP23 | 0.4217 | 0.0504 | 0.7084 | 0.4971 | 0.6312 | 0.4737 | 0.7918 | 0.4631 |
|  | SOST | 2.9711 | 0.6094 | 3.2138 | 0.6968 | 2.8482 | 0.4740 | 2.4681 | 0.4297 |
|  | TYMP | 5.2966 | 0.4622 | 5.7831 | 0.2879 | 5.3607 | 0.5508 | 5.1209 | 0.3774 |

Supplementary Table S5 Biomarker effect sizes between groups (H - healthy, CD - Crohn's disease, UC - ulcerative colitis, IBS - irritable bowel syndrome, IBD - inflammatory bowel disease)

|  |  | **CD vs H** | **UC vs H** | **IBS vs H** | **CD vs UC** | **UC vs IBS** | **CD vs IBS** | **IBD vs H** | **IBD vs IBS** |
| --- | --- | --- | --- | --- | --- | --- | --- | --- | --- |
| inflammation panel | AXIN1 | 0.7756 | 0.8141 | 0.8116 | 0.2012 | 0.0100 | 0.1706 | 0.7949 | 0.0903 |
|  | CASP-8 | 0.7051 | 0.6410 | 0.4275 | 0.1006 | 0.2107 | 0.2642 | 0.6731 | 0.2375 |
|  | CCL11 | 0.0641 | 0.1923 | -0.3044 | -0.0888 | 0.5251 | 0.2977 | 0.1282 | 0.4114 |
|  | CCL19 | 0.3077 | 0.2308 | -0.0725 | 0.1479 | 0.3311 | 0.5117 | 0.2692 | 0.4214 |
|  | CCL20 | 0.5513 | 0.5897 | 0.0580 | 0.0178 | 0.5050 | 0.4716 | 0.5705 | 0.4883 |
|  | CCL28 | 0.0128 | -0.1282 | -0.5145 | 0.1598 | 0.5987 | 0.6388 | -0.0577 | 0.6187 |
|  | CD5 | 0.0897 | 0.1410 | -0.1522 | 0.0533 | 0.4247 | 0.4047 | 0.1154 | 0.4147 |
|  | CD6 | 0.0513 | -0.1410 | -0.3841 | 0.1716 | 0.3579 | 0.5117 | -0.0449 | 0.4348 |
|  | CDCP1 | 0.4359 | 0.1282 | -0.1015 | 0.3373 | 0.2776 | 0.4983 | 0.2821 | 0.3880 |
|  | CXCL10 | 0.0897 | -0.1795 | -0.6232 | 0.2781 | 0.3913 | 0.6923 | -0.0449 | 0.5418 |
|  | CXCL11 | 0.5000 | 0.1538 | -0.1232 | 0.2544 | 0.2441 | 0.5518 | 0.3269 | 0.3980 |
|  | CXCL9 | 0.4359 | 0.3333 | -0.4493 | 0.0533 | 0.6388 | 0.7458 | 0.3846 | 0.6923 |
|  | EN-RAGE | 0.7692 | 0.8205 | 0.6957 | 0.2544 | -0.0836 | 0.0970 | 0.7949 | 0.0067 |
|  | FGF-19 | -0.7308 | -0.2949 | -0.5145 | -0.4083 | 0.1839 | -0.2709 | -0.5128 | -0.0435 |
|  | FGF-23 | 0.2821 | -0.0513 | -0.3152 | 0.3965 | 0.1906 | 0.5853 | 0.1154 | 0.3880 |
|  | HGF | 0.7051 | 0.2564 | 0.1812 | 0.3373 | 0.1104 | 0.4716 | 0.4808 | 0.2910 |
|  | IL-17A | 0.3974 | 0.5513 | 0.0652 | -0.1953 | 0.8395 | 0.5518 | 0.4744 | 0.6957 |
|  | IL-10 | -0.3846 | -0.2821 | -0.7681 | -0.2308 | 0.5385 | 0.2776 | -0.3333 | 0.4080 |
|  | IL-18 | 0.1282 | -0.2692 | -0.4565 | 0.4201 | 0.1706 | 0.5385 | -0.0705 | 0.3545 |
|  | IL-6 | 0.2051 | -0.4487 | -0.5217 | 0.4556 | 0.0936 | 0.5050 | -0.1218 | 0.2993 |
|  | LIF-R | 0.3205 | 0.1282 | -0.1304 | 0.0651 | 0.3579 | 0.5050 | 0.2244 | 0.4314 |
|  | OPG | -0.2051 | -0.3974 | -0.5435 | 0.3018 | -0.0368 | 0.4047 | -0.3013 | 0.1839 |
|  | OSM | 0.9231 | 0.7436 | 0.6449 | 0.3254 | 0.1104 | 0.4448 | 0.8333 | 0.2776 |
|  | PD-L1 | -0.0641 | -0.3718 | -0.4928 | 0.2781 | 0.0769 | 0.4649 | -0.2180 | 0.2709 |
|  | SIRT-2 | 0.5385 | 0.5769 | 0.5942 | 0.0414 | 0.0569 | 0.0702 | 0.5577 | 0.0635 |
|  | ST1A1 | 0.8718 | 0.7051 | 0.7609 | 0.3728 | -0.3445 | 0.0234 | 0.7885 | -0.1605 |
|  | TGF-α | 0.7949 | 0.4872 | 0.4638 | 0.2426 | 0.0502 | 0.3779 | 0.6410 | 0.2140 |
|  | TNFRSF9 | 0.1795 | 0.1923 | -0.4710 | 0.0651 | 0.7191 | 0.6455 | 0.1859 | 0.6823 |
|  | TNFSF14 | 0.9487 | 0.7821 | 0.8913 | 0.3136 | 0.0635 | 0.3512 | 0.8654 | 0.2074 |
|  | VEGFA | 0.5641 | 0.2821 | -0.0145 | 0.3491 | 0.2441 | 0.5518 | 0.4231 | 0.3980 |
| metabolism panel | ACP6 | -0.6410 | -0.5897 | -0.5797 | -0.2308 | 0.0368 | -0.2241 | -0.6154 | -0.0937 |
|  | ADGRE2 | -0.4359 | -0.4359 | -0.7029 | 0.1479 | 0.0301 | 0.1505 | -0.4359 | 0.0903 |
|  | ADGRG2 | -0.8077 | -0.4872 | -0.4203 | -0.1124 | -0.1438 | -0.2843 | -0.6474 | -0.2141 |
|  | ANGPT2 | 0.3974 | 0.3846 | 0.0145 | -0.1479 | 0.4582 | 0.4114 | 0.3910 | 0.4348 |
|  | APEX1 | 0.6923 | 0.5385 | 0.4819 | 0.2544 | -0.0769 | 0.1906 | 0.6154 | 0.0569 |
|  | CA13 | 0.6923 | 0.8590 | 0.7826 | 0.1124 | 0.0100 | 0.0702 | 0.7756 | 0.0401 |
|  | CCDC80 | -0.5256 | -0.6410 | -0.6739 | -0.0178 | 0.0970 | 0.1037 | -0.5833 | 0.1003 |
|  | CD164 | 0.5385 | 0.6538 | 0.3913 | 0.1124 | 0.0769 | 0.1572 | 0.5962 | 0.1171 |
|  | CD79B | 0.3846 | 0.1282 | -0.0797 | 0.2781 | 0.2843 | 0.6187 | 0.2564 | 0.4515 |
|  | CHRDL2 | -0.0513 | -0.3205 | -0.5942 | 0.1953 | 0.2642 | 0.4247 | -0.1859 | 0.3445 |
|  | CLEC5A | 0.6410 | 0.6410 | 0.3406 | 0.1834 | 0.1237 | 0.2575 | 0.6410 | 0.1906 |
|  | DPP7 | 0.6026 | 0.5385 | 0.6377 | 0.2781 | -0.2709 | 0.0502 | 0.5705 | -0.1104 |
|  | FAM3C | -0.3462 | -0.4487 | -0.5797 | 0.1243 | 0.0368 | 0.2575 | -0.3974 | 0.1472 |
|  | GAL | -0.6539 | -0.5641 | -0.0435 | -0.1834 | -0.4916 | -0.5786 | -0.6090 | -0.5351 |
|  | GRAP2 | 0.5897 | 0.6282 | 0.4058 | 0.0000 | 0.1706 | 0.1572 | 0.6090 | 0.1639 |
|  | LILRA5 | 0.7308 | 0.4487 | 0.3261 | 0.3254 | 0.0502 | 0.3512 | 0.5897 | 0.2007 |
|  | NADK | 0.6923 | 0.8590 | 0.7971 | 0.1361 | -0.1973 | -0.0167 | 0.7756 | -0.1070 |
|  | PILRB | 0.3590 | 0.1795 | -0.2246 | 0.2663 | 0.4381 | 0.5452 | 0.2692 | 0.4916 |
|  | PPP1R2 | 0.6154 | 0.5897 | 0.4239 | 0.1953 | 0.1371 | 0.1906 | 0.6026 | 0.1639 |
|  | RNASE3 | 0.8974 | 0.7821 | 0.7101 | 0.2781 | 0.1773 | 0.4515 | 0.8397 | 0.3144 |
|  | ROR1 | -0.7821 | -0.6154 | -0.6739 | -0.3728 | 0.1773 | -0.1572 | -0.6987 | 0.0100 |
|  | SIGLEC7 | -0.1795 | -0.4359 | -0.6232 | 0.2663 | 0.2308 | 0.5117 | -0.3077 | 0.3712 |
|  | SNAP23 | 0.6410 | 0.7244 | 0.6812 | 0.0178 | 0.0167 | 0.0033 | 0.6827 | 0.0100 |
|  | SOST | 0.1410 | -0.1154 | -0.5217 | 0.2544 | 0.4582 | 0.4716 | 0.0128 | 0.4649 |
|  | TYMP | 0.6667 | 0.1667 | -0.0217 | 0.4675 | 0.2977 | 0.7592 | 0.4167 | 0.5284 |

Supplementary Table S6 Bacterial families in faecal samples, effect sizes between groups. The 35 differentially abundant bacterial families present in all subject groups, with the effect sizes >0.3 in any of the group comparisons, chosen for the association with serum protein data, are listed above the demarcation line. (H - healthy, CD - Crohn's disease, UC - ulcerative colitis, IBS - irritable bowel syndrome, IBD - inflammatory bowel disease)

| **family** | **CD vs H** | **UC vs H** | **IBS vs H** | **CD vs UC** | **UC vs IBS** | **CD vs IBS** | **IBD vs H** | **IBD vs IBS** |
| --- | --- | --- | --- | --- | --- | --- | --- | --- |
| [Barnesiellaceae] | -0.0350 | 0.1826 | 0.5215 | -0.0948 | -0.2804 | -0.4174 | 0.0385 | -0.3082 |
| [Cerasicoccaceae] | 0.2263 | 0.6190 | 0.4845 | -0.2626 | 0.1337 | -0.2914 | 0.3830 | -0.0303 |
| [Mogibacteriaceae] | 0.3841 | 0.4948 | 0.2396 | 0.0074 | 0.2111 | 0.1337 | 0.3939 | 0.1612 |
| Aerococcaceae | 0.2709 | 0.3011 | 0.2624 | 0.0392 | 0.1345 | 0.0810 | 0.2653 | 0.0565 |
| Alcaligenaceae | -0.2061 | -0.1534 | -0.3021 | 0.0203 | 0.0304 | 0.0283 | -0.3308 | -0.0818 |
| Bacteroidaceae | -0.2717 | -0.4049 | -0.2074 | 0.3420 | -0.0790 | 0.0896 | -0.4345 | -0.1149 |
| Bifidobacteriaceae | 0.2372 | 0.4070 | 0.0778 | -0.1513 | 0.3847 | 0.3014 | 0.2418 | 0.2528 |
| Campylobacteraceae | -0.0066 | 0.3279 | 0.2598 | -0.3178 | 0.1421 | -0.2056 | 0.1524 | -0.0313 |
| Christensenellaceae | 0.5526 | 0.6850 | 0.4108 | -0.0233 | 0.2850 | 0.0731 | 0.5344 | 0.1649 |
| Coriobacteriaceae | 0.1874 | 0.3553 | 0.0673 | -0.0980 | 0.4743 | 0.2760 | 0.1825 | 0.1725 |
| Dehalobacteriaceae | 0.5052 | 0.6706 | 0.4479 | -0.1017 | 0.1652 | 0.0590 | 0.5564 | 0.1105 |
| Desulfovibrionaceae | 0.5019 | 0.4676 | 0.1850 | 0.0711 | 0.3352 | 0.2868 | 0.3586 | 0.2431 |
| Elusimicrobiaceae | 0.4091 | 0.2948 | 0.3045 |  | 0.0826 | 0.0618 | 0.3012 | 0.0600 |
| Enterobacteriaceae | -0.7250 | -0.4372 | -0.4253 | -0.2543 | 0.0490 | -0.2704 | -0.4970 | -0.0768 |
| Erysipelotrichaceae | -0.3855 | -0.2507 | -0.0606 | -0.2608 | -0.0013 | -0.2477 | -0.4152 | -0.1500 |
| EtOH8 | 0.4959 | 0.4214 | 0.4876 | 0.0972 | -0.0541 | 0.0451 | 0.3613 | -0.0315 |
| Eubacteriaceae | 0.0214 | 0.3369 | 0.1527 | -0.2719 | 0.2370 | -0.0453 | 0.1228 | 0.0501 |
| Fusobacteriaceae | -0.1433 | -0.3609 | -0.1078 | 0.1183 | -0.2065 | -0.0163 | -0.2847 | -0.1405 |
| Lactobacillaceae | -0.3052 | -0.3345 | -0.1553 | -0.0872 | -0.2100 | -0.1437 | -0.3534 | -0.2266 |
| Leuconostocaceae | 0.3347 | 0.1206 | 0.0820 | 0.1247 | 0.1457 | 0.2303 | 0.1784 | 0.1589 |
| Methanobacteriaceae | 0.3313 | 0.4181 | 0.3640 | -0.0557 | 0.0618 | 0.0078 | 0.3423 | 0.0598 |
| Moraxellaceae | 0.3649 | 0.2635 | 0.2302 | 0.0874 | 0.0485 | 0.1410 | 0.3145 | 0.0733 |
| Neisseriaceae | 0.1028 | 0.0388 | 0.3366 | 0.0821 | -0.2724 | -0.0947 | 0.0545 | -0.1398 |
| Pasteurellaceae | -0.3444 | -0.5820 | 0.0390 | 0.3092 | -0.5442 | -0.3609 | -0.4580 | -0.4326 |
| Peptococcaceae | 0.1388 | 0.3166 | 0.3299 | -0.1086 | -0.0336 | -0.1654 | 0.1518 | -0.0719 |
| Rikenellaceae | 0.1578 | 0.3280 | 0.3073 | -0.0259 | 0.1751 | 0.0504 | 0.1323 | 0.0075 |
| Ruminococcaceae | 0.4655 | 0.5461 | 0.5117 | -0.0515 | 0.2084 | -0.0137 | 0.4868 | -0.0091 |
| S24-7 | 0.2388 | -0.2773 | -0.0558 | 0.4588 | -0.1786 | 0.3241 | 0.0245 | 0.0907 |
| Staphylococcaceae | 0.2283 | 0.3645 | 0.2038 | -0.0781 | 0.0768 | 0.0283 | 0.2521 | 0.0648 |
| Streptococcaceae | -0.1151 | -0.3836 | -0.0455 | 0.2966 | -0.2449 | 0.0053 | -0.2519 | -0.1963 |
| Synergistaceae | 0.4223 | 0.1715 | 0.2028 | 0.2340 | -0.0153 | 0.2930 | 0.2421 | 0.0749 |
| Turicibacteraceae | 0.1652 | -0.3956 | -0.0208 | 0.5083 | -0.2937 | 0.1321 | -0.1025 | -0.0815 |
| Veillonellaceae | 0.6834 | 0.0464 | 0.3313 | 0.6517 | -0.1179 | 0.3955 | 0.1040 | -0.0006 |
| Verrucomicrobiaceae | 0.3737 | 0.6684 | 0.2977 | -0.2210 | 0.2481 | -0.0085 | 0.4677 | 0.1063 |
| Victivallaceae | 0.3793 | 0.3609 | 0.4986 | 0.0021 | -0.0773 | -0.0447 | 0.4151 | -0.0479 |
| [Methanomassiliicoccaceae] | 0.3236 | 0.1563 | 0.2681 | 0.1837 | -0.1114 |  | 0.2914 | 0.0024 |
| [Odoribacteraceae] | 0.0814 | 0.0686 | 0.2605 | 0.0520 | -0.0713 | -0.0616 | 0.0986 | -0.0910 |
| [Paraprevotellaceae] | 0.0602 | 0.0121 | -0.2135 | 0.0433 | 0.1203 | 0.2271 | 0.0019 | 0.1584 |
| [Tissierellaceae] | -0.0252 | 0.0223 | 0.0779 | -0.0786 | 0.0043 | -0.0354 | -0.0314 | -0.0749 |
| Actinomycetaceae | -0.0472 | -0.0957 | -0.0450 | 0.0877 | 0.0123 | 0.1082 | -0.1832 | -0.0278 |
| Aeromonadaceae | 0.1559 | 0.2300 |  | -0.0170 | -0.0354 | -0.0719 | 0.1931 | -0.0033 |
| Anaerolinaceae | 0.1935 |  |  | -0.0315 | 0.0000 | -0.0029 | 0.1925 | -0.0170 |
| Anaeroplasmataceae |  | 0.1771 |  | 0.1117 | -0.0187 |  | 0.1913 | -0.0340 |
| Bacillaceae | 0.2235 | 0.2771 | 0.2819 | 0.0741 | 0.0254 | -0.0140 | 0.2121 | -0.0199 |
| Bdellovibrionaceae |  |  | 0.2366 |  | 0.0552 | 0.0174 |  | 0.0623 |
| Burkholderiaceae | 0.1823 |  | 0.2304 | -0.0077 | 0.0485 | 0.0260 | 0.1616 | 0.0215 |
| Cardiobacteriaceae | 0.1515 | 0.2195 |  | -0.0280 | -0.0229 | -0.1000 | 0.1435 | -0.0440 |
| Carnobacteriaceae | -0.0332 | 0.0704 | 0.0935 | -0.0654 | -0.0081 | -0.1287 | 0.0133 | -0.0711 |
| Clostridiaceae | 0.2128 | 0.2782 | 0.0529 | -0.0061 | 0.2042 | 0.2624 | 0.1618 | 0.1132 |
| Comamonadaceae | 0.2989 | 0.2123 | 0.1561 | 0.1062 | 0.0558 | 0.1459 | 0.2272 | 0.0423 |
| Corynebacteriaceae | 0.2263 | 0.1553 | 0.2081 | 0.1289 | -0.0311 | 0.0400 | 0.0859 | -0.0249 |
| Deinococcaceae | 0.2146 | 0.2312 | 0.2728 | 0.0461 | 0.0366 | 0.0595 | 0.2295 | -0.0005 |
| Dethiosulfovibrionaceae |  | 0.2117 | 0.2121 | 0.1018 | 0.0293 | 0.0947 | 0.2239 | 0.0412 |
| Enterococcaceae | 0.1463 | -0.0637 | 0.0160 | 0.2088 | -0.0061 | 0.1753 | 0.0056 | 0.0292 |
| F16 | 0.2366 | 0.1972 | 0.1981 | 0.0523 | 0.0494 | 0.0678 | 0.2210 | 0.0450 |
| Gemellaceae | -0.0647 | -0.0326 | 0.1721 | -0.0710 | -0.1548 | -0.2690 | -0.0833 | -0.2369 |
| Gracilibacteraceae |  |  | 0.1989 |  | 0.0640 | 0.1010 |  | 0.0600 |
| Helicobacteraceae | 0.2608 | 0.2171 | 0.2889 | 0.1020 | -0.0332 | 0.0558 | 0.2027 | -0.0050 |
| Lachnospiraceae | 0.0252 | 0.2768 | 0.1902 | -0.1745 | 0.2466 | -0.1033 | -0.1120 | -0.0179 |
| Leptospiraceae | 0.2128 |  |  | -0.0622 | 0.0000 | 0.0185 | 0.2429 | -0.0136 |
| Leptotrichiaceae |  | 0.1468 |  | 0.1078 | -0.0290 |  | 0.2116 | -0.0552 |
| Listeriaceae | 0.2381 | 0.2820 | 0.2766 | 0.0686 | 0.0087 | 0.0084 | 0.2615 | 0.0018 |
| Micrococcaceae | -0.1145 | 0.1060 | 0.0753 | -0.1854 | 0.0637 | -0.1815 | -0.0601 | -0.0711 |
| Nitrosomonadaceae | 0.2271 |  |  | 0.0139 | 0.0000 | -0.0097 | 0.2607 | -0.0262 |
| Oxalobacteraceae | 0.1891 | 0.0967 | 0.1495 | 0.1426 | 0.0000 | 0.1295 | 0.0998 | -0.0264 |
| Paenibacillaceae |  |  | 0.2320 |  | 0.0634 | 0.0830 |  | 0.0540 |
| Peptostreptococcaceae | 0.0236 | -0.1690 | 0.0576 | 0.1456 | -0.1532 | 0.1074 | -0.0476 | -0.0123 |
| Phyllobacteriaceae | 0.2601 |  |  | 0.0218 | 0.0000 | 0.0087 | 0.1994 | -0.0136 |
| Planococcaceae | 0.3360 | 0.3337 | 0.2923 |  | 0.0000 |  | 0.2594 | -0.0217 |
| Porphyromonadaceae | -0.1144 | 0.1883 | -0.0110 | -0.1562 | 0.2935 | -0.0430 | -0.0473 | 0.1215 |
| Prevotellaceae | 0.0154 | -0.0875 | -0.1568 | 0.1058 | 0.0811 | 0.2652 | -0.0669 | 0.1140 |
| Propionibacteriaceae |  | 0.1112 |  | 0.1716 | -0.1261 |  | 0.2135 | -0.0566 |
| Pseudomonadaceae | 0.2422 | 0.2780 | 0.2965 | 0.0190 | -0.0151 | -0.0647 | 0.2407 | -0.0333 |
| Rhodobacteraceae | 0.2667 | 0.2987 | 0.2959 | 0.0321 | -0.0289 | -0.0594 | 0.2254 | -0.0326 |
| Rhodocyclaceae |  | 0.2195 | 0.2104 | 0.0556 | 0.0202 | 0.1005 | 0.2019 | 0.0444 |
| Succinivibrionaceae | 0.0652 | 0.0148 | 0.1423 | -0.0426 | -0.0623 | 0.0457 | 0.0589 | -0.0336 |
| Syntrophomonadaceae |  |  | 0.1776 |  | 0.1226 | 0.1140 |  | 0.1180 |
| Xanthomonadaceae |  |  | 0.2485 |  | 0.0822 | 0.0503 |  | 0.0546 |

Supplementary Table S7 Biomarker Youden index (J) and AUC values between groups (H - healthy, CD - Crohn's disease, UC - ulcerative colitis, IBS - irritable bowel syndrome, IBD - inflammatory bowel disease)

|  |  | **H vs CD** | | **H vs UC** | | **H vs IBS** | | **UC vs CD** | | **UC vs IBS** | | **CD vs IBS** | | **H vs IBD** | | **IBD vs IBS** | |
| --- | --- | --- | --- | --- | --- | --- | --- | --- | --- | --- | --- | --- | --- | --- | --- | --- | --- |
|  |  | **J** | **AUC** | **J** | **AUC** | **J** | **AUC** | **J** | **AUC** | **J** | **AUC** | **J** | **AUC** | **J** | **AUC** | **J** | **AUC** |
| inflammation panel | AXIN1 | 0.769 | 0.888 | 0.786 | 0.916 | 0.783 | 0.906 | 0.264 | 0.569 | 0.177 | 0.528 | 0.268 | 0.585 | 0.778 | 0.901 | 0.145 | 0.556 |
|  | CASP-8 | 0.679 | 0.853 | 0.619 | 0.821 | 0.442 | 0.715 | 0.256 | 0.571 | 0.304 | 0.59 | 0.324 | 0.632 | 0.648 | 0.836 | 0.267 | 0.61 |
|  | CCL20 | 0.583 | 0.776 | 0.607 | 0.798 | 0.272 | 0.529 | 0.247 | 0.505 | 0.438 | 0.761 | 0.435 | 0.736 | 0.583 | 0.787 | 0.435 | 0.749 |
|  | CCL28 | 0.179 | 0.506 | 0.298 | 0.565 | 0.54 | 0.757 | 0.308 | 0.588 | 0.584 | 0.804 | 0.572 | 0.819 | 0.176 | 0.531 | 0.573 | 0.812 |
|  | CXCL10 | 0.186 | 0.545 | 0.333 | 0.565 | 0.572 | 0.812 | 0.423 | 0.615 | 0.429 | 0.717 | 0.706 | 0.846 | 0.13 | 0.512 | 0.51 | 0.779 |
|  | CXCL9 | 0.538 | 0.718 | 0.5 | 0.69 | 0.446 | 0.725 | 0.143 | 0.516 | 0.553 | 0.832 | 0.619 | 0.873 | 0.519 | 0.704 | 0.585 | 0.852 |
|  | EN-RAGE | 0.756 | 0.885 | 0.833 | 0.905 | 0.703 | 0.848 | 0.346 | 0.643 | 0.22 | 0.55 | 0.237 | 0.548 | 0.796 | 0.895 | 0.176 | 0.502 |
|  | IL-17A | 0.442 | 0.699 | 0.679 | 0.78 | 0.232 | 0.533 | 0.313 | 0.615 | 0.742 | 0.925 | 0.615 | 0.776 | 0.565 | 0.741 | 0.66 | 0.853 |
|  | IL-10 | 0.455 | 0.692 | 0.381 | 0.595 | 0.699 | 0.884 | 0.401 | 0.643 | 0.568 | 0.768 | 0.308 | 0.639 | 0.37 | 0.642 | 0.412 | 0.715 |
|  | OSM | 0.923 | 0.962 | 0.786 | 0.881 | 0.739 | 0.822 | 0.401 | 0.659 | 0.28 | 0.565 | 0.441 | 0.722 | 0.852 | 0.92 | 0.343 | 0.641 |
|  | ST1A1 | 0.769 | 0.936 | 0.643 | 0.863 | 0.739 | 0.88 | 0.335 | 0.654 | 0.366 | 0.643 | 0.184 | 0.512 | 0.704 | 0.898 | 0.208 | 0.568 |
|  | TNFRSF9 | 0.269 | 0.59 | 0.345 | 0.607 | 0.457 | 0.736 | 0.198 | 0.511 | 0.627 | 0.866 | 0.726 | 0.823 | 0.306 | 0.599 | 0.654 | 0.845 |
|  | TNFSF14 | 0.846 | 0.974 | 0.762 | 0.899 | 0.75 | 0.946 | 0.319 | 0.643 | 0.211 | 0.55 | 0.378 | 0.676 | 0.796 | 0.935 | 0.275 | 0.61 |
| metabolism panel | ACP6 | 0.679 | 0.821 | 0.548 | 0.798 | 0.623 | 0.79 | 0.275 | 0.588 | 0.183 | 0.506 | 0.281 | 0.612 | 0.611 | 0.809 | 0.09 | 0.557 |
|  | CA13 | 0.692 | 0.846 | 0.857 | 0.935 | 0.739 | 0.891 | 0.187 | 0.527 | 0.121 | 0.534 | 0.234 | 0.535 | 0.778 | 0.892 | 0.147 | 0.535 |
|  | CCDC80 | 0.526 | 0.763 | 0.679 | 0.821 | 0.62 | 0.837 | 0.313 | 0.522 | 0.236 | 0.565 | 0.191 | 0.552 | 0.574 | 0.793 | 0.187 | 0.559 |
|  | DPP7 | 0.519 | 0.801 | 0.583 | 0.786 | 0.54 | 0.819 | 0.33 | 0.621 | 0.382 | 0.606 | 0.254 | 0.525 | 0.546 | 0.793 | 0.258 | 0.543 |
|  | GAL | 0.526 | 0.827 | 0.512 | 0.768 | 0.192 | 0.522 | 0.385 | 0.615 | 0.425 | 0.724 | 0.508 | 0.789 | 0.509 | 0.796 | 0.449 | 0.755 |
|  | NADK | 0.686 | 0.844 | 0.833 | 0.929 | 0.746 | 0.899 | 0.308 | 0.582 | 0.379 | 0.606 | 0.244 | 0.508 | 0.759 | 0.889 | 0.262 | 0.559 |
|  | RNASE3 | 0.917 | 0.949 | 0.845 | 0.899 | 0.786 | 0.855 | 0.253 | 0.604 | 0.248 | 0.618 | 0.401 | 0.726 | 0.88 | 0.923 | 0.293 | 0.67 |
|  | ROR1 | 0.763 | 0.891 | 0.607 | 0.805 | 0.576 | 0.837 | 0.401 | 0.703 | 0.335 | 0.606 | 0.281 | 0.579 | 0.657 | 0.846 | 0.145 | 0.517 |
|  | SNAP23 | 0.609 | 0.821 | 0.702 | 0.872 | 0.656 | 0.841 | 0.242 | 0.5 | 0.149 | 0.522 | 0.167 | 0.502 | 0.657 | 0.847 | 0.079 | 0.512 |
|  | TYMP | 0.583 | 0.833 | 0.274 | 0.583 | 0.239 | 0.511 | 0.489 | 0.742 | 0.382 | 0.655 | 0.739 | 0.88 | 0.361 | 0.704 | 0.554 | 0.763 |
| bacterial family | Christensenellaceae | 0.609 | 0.776 | 0.609 | 0.833 | 0.542 | 0.767 | 0.154 | 0.574 | 0.301 | 0.619 | 0.224 | 0.534 | 0.609 | 0.804 | 0.263 | 0.576 |
|  | Enterobacteriaceae | 0.442 | 0.679 | 0.372 | 0.647 | 0.375 | 0.622 | 0.231 | 0.556 | 0.173 | 0.5 | 0.231 | 0.571 | 0.404 | 0.663 | 0.154 | 0.535 |
|  | Erysipellotrichaceae | 0.449 | 0.667 | 0.25 | 0.513 | 0.25 | 0.538 | 0.385 | 0.692 | 0.17 | 0.519 | 0.298 | 0.644 | 0.295 | 0.59 | 0.157 | 0.562 |
|  | Pasteurellaceae | 0.429 | 0.721 | 0.609 | 0.843 | 0.333 | 0.635 | 0.308 | 0.63 | 0.506 | 0.753 | 0.346 | 0.631 | 0.474 | 0.782 | 0.391 | 0.692 |
|  | Ruminococcaceae | 0.519 | 0.821 | 0.603 | 0.846 | 0.458 | 0.781 | 0.308 | 0.598 | 0.26 | 0.583 | 0.304 | 0.545 | 0.526 | 0.833 | 0.144 | 0.519 |
|  | Turicibacteraceae | 0.353 | 0.561 | 0.417 | 0.515 | 0.208 | 0.523 | 0.615 | 0.769 | 0.381 | 0.631 | 0.311 | 0.591 | 0.263 | 0.527 | 0.189 | 0.52 |
|  | Veillonellaceae | 0.442 | 0.731 | 0.212 | 0.506 | 0.333 | 0.649 | 0.385 | 0.669 | 0.288 | 0.622 | 0.221 | 0.561 | 0.288 | 0.612 | 0.151 | 0.53 |
|  | Verrucomicrobiaceae | 0.359 | 0.622 | 0.513 | 0.769 | 0.375 | 0.663 | 0.385 | 0.675 | 0.266 | 0.615 | 0.144 | 0.546 | 0.436 | 0.696 | 0.183 | 0.534 |

Supplementary Table S8 CRP levels, with median values and standard deviations. Statistical significance of Wilcoxon paired test is given as FDR-corrected p-value. Youden index (J) and AUC values between groups (CD - Crohn's disease, UC - ulcerative colitis, IBS - irritable bowel syndrome, IBD - inflammatory bowel disease)

| CRP (mg/L) per study group | | | | |
| --- | --- | --- | --- | --- |
|  | CD | UC | IBS | IBD |
| median | 2.2 | 0.7 | 0.6 | 1.9 |
| SD | 10.2 | 4.8 | 4.4 | 8.0 |
| Comparison of CRP between groups | | | | |
|  | IBD vs IBS | CD vs IBS | CD vs UC | UC vs IBS |
| p | 0.013 | 0.007 | 0.057 | 0.266 |
| Youden J | 0.365 | 0.596 | 0.538 | 0.246 |
| AUC | 0.690 | 0.790 | 0.725 | 0.590 |
